# Tumor cells that resist neutrophil anticancer cytotoxicity acquire a prometastatic and innate immune escape phenotype

**DOI:** 10.1038/s41423-025-01283-w

**Published:** 2025-03-28

**Authors:** Jagoda Agnieszka Szlachetko, Francisca Hofmann-Vega, Bettina Budeus, Lara-Jasmin Schröder, Claudia Alexandra Dumitru, Mathias Schmidt, Eric Deuss, Sebastian Vollmer, Eva-Maria Hanschmann, Maike Busch, Jan Kehrmann, Stephan Lang, Nicole Dünker, Timon Hussain, Sven Brandau

**Affiliations:** 1 Department of Otorhinolaryngology, University Hospital Essen, Essen, 45147 Germany; 2 Institute of Cell Biology, University Hospital Essen, Essen, 45147 Germany; 3 Institute of Pathology, Medical School Hannover, Hannover, 30625 Germany; 4 Department of Neurosurgery, Otto-von-Guericke University, Magdeburg, 39106 Germany; 5 Institute of Anatomy II, Department of Neuroanatomy, Center for Translational Neuro- and Behavioral Sciences (C-TNBS), University of Duisburg-Essen, Medical Faculty, Essen, 45147 Germany; 6 Institute of Medical Microbiology, University Hospital Essen, Essen, 45147 Germany; 7 Department of Otorhinolaryngology, Klinikum rechts der Isar, Technical University Munich, Munich, 81675 Germany; 8 German Cancer Consortium, DKTK, Partner Site Essen-Düsseldorf, Essen, 45147 Germany

**Keywords:** Lymphatic metastasis, Tumor-associated neutrophils, Dysbiosis and oral microbiome, neutrophil elastase, Neutrophil extracellular traps, Epithelial‒mesenchymal transition, *Staphylococcus aureus*, Head and neck cancer, Immune evasion, Tumour immunology, Cancer microenvironment

## Abstract

In the tumor host, neutrophils may exhibit protumor or antitumor activity. It is hypothesized that in response to host-derived or therapy-induced factors, neutrophils adopt diverse functional states to ultimately execute these differential functions. Here, we provide an alternative scenario in which the response of an individual tumor cell population determines the overall protumor versus antitumor outcome of neutrophil‒tumor interactions. Experimentally, we show that human neutrophils, which are sequentially stimulated with bacteria and secreted factors from tumor cells, kill a certain proportion of tumor target cells. However, the majority of the tumor cells remained resistant to this neutrophil-mediated killing and underwent a functional, phenotypic and transcriptomic switch that was reminiscent of partial epithelial‒to-mesenchymal transition. This cell biological switch was associated with physical escape from NK-mediated killing and resulted in enhanced metastasis to the lymph nodes in a preclinical orthotopic mouse model. Mechanistically, we identified the antimicrobial neutrophil granule proteins neutrophil elastase (NE) and matrix metalloprotease-9 (MMP-9) as the molecular mediators of this functional switch. We validated these data in patients with head and neck cancer and identified bacterially colonized intratumoral niches that were enriched for mesenchymal tumor cells and neutrophils expressing NE and MMP-9. Our data reveal the parallel execution of tumor cytotoxic and prometastatic activity by activated neutrophils and identify NE and MMP-9 as mediators of lymph node metastasis. The identified mechanism explains the functional dichotomy of tumor-associated neutrophils at the level of the tumor target cell response and has implications for superinfected cancers and the dysbiotic tumor microenvironment.

## Introduction

Neutrophils are primary innate immune cells that play a major role in the defense against pathogens. As such, they are equipped with an arsenal of antibacterial compounds, many of which are stored in their granules. These include myeloperoxidase (MPO) and neutrophil elastase (NE), which are localized in azurophilic granules, as well as matrix metalloproteinase-9 (MMP-9) stored in gelatinase granules. Many granule components are important neutrophil effector molecules during bacterial infection, but they also function as mediators of tumor progression and development in tumor-associated neutrophils (TANs) [1].

Specifically, NE was found to induce tumor cell proliferation and angiogenesis [2–4]. NE also activates MMP-9 via inactivation of natural gelatinase inhibitors (TIMPs) [4] and thus indirectly influences the migratory abilities of tumor cells [5]. More recently, it was shown that human neutrophils from various sources of healthy donors and cancer patients release soluble NE that is equipped with direct antitumor properties [6]. The authors demonstrated that human, but not murine, NE was the major anticancer protein in human neutrophil media and killed cancer cells via a mechanism that involved proteolytic liberation of the CD95 death domain in target cells [6].

NE and MMP-9 are also the main components of web-like structures of extracellular nuclear and mitochondrial DNA that are expelled from activated neutrophils [7]. These structures are known as neutrophil extracellular traps (NETs) and primarily appear to have antipathogenic functions, but in the context of cancer, NETs can also promote tumor metastasis [8–10]. Thus, multiple antimicrobial features of neutrophils appear to be hijacked by the tumor microenvironment to mediate various mechanisms that collaboratively promote carcinogenesis and tumor progression.

This “dual use” of the antimicrobial arsenal of neutrophils is highly relevant because many tumors in the human body arise at surfaces such as the skin, oral cavity, and respiratory or gastrointestinal tract, all of which are colonized by bacteria [11]. At these sites, local progressive tumor growth or therapy-induced tissue disintegration may cause barrier defects, dysbiosis or superinfection. Therefore, we hypothesized that bacterial stimulation may modulate the cross-talk of tumor cells and neutrophils in the TME. To test this hypothesis, we developed an in vitro system that mimics this cross-talk between tumor cells and neutrophils and exposed this system to bacterial stimulation. As model organisms, we used bacteria relevant to mucosal barrier organs; as model cell lines, we used carcinomas arising in the oropharyngeal cavity and the respiratory tract.

In recent years, the heterogeneity, plasticity, and functional dichotomy of neutrophils in cancer has generated broad interest in the field [12–15]. Multiple scenarios and models in which TANs can have either protumor or antitumor properties have been described [8–10, 16–23]. According to current views in the field, this dichotomy is explained by the capacity of neutrophils to acquire different functional states, which can lead to several protumor or antitumor phenotypes [24–28]. Examples include antitumor neutrophils with T-cell stimulatory and antigen-presenting capacities [18, 28], neutrophils with tumor-cytotoxic functions [29], interferon-stimulated neutrophils with broad antitumor functions [30] or various proangiogenic and prometastatic phenotypes [24, 27, 28, 31].

In this work, we aim to provide an alternative explanation for the functional TAN dichotomy in the context of cancer biology. In our models, the functional dichotomy is explained by differential responses of tumor target cells and thus does not depend primarily on neutrophil functional heterogeneity but rather on the dichotomous susceptibility and response of the tumor target cells themselves. In several model systems involving neutrophil activation by bacteria and tumor cells, we observed that stimulated neutrophils can kill a proportion of tumor target cells and thus have direct antitumor activity. Importantly, tumor cells that survive neutrophil attack acquire a prometastatic and innate immune escape phenotype when exposed to factors secreted by stimulated neutrophils.

Since many tumors that are exposed to the outer environment of the body are either colonized by bacteria or prone to superinfection [11, 32–40], our findings not only provide insights into the functional dichotomy of TANs but also have biological implications for dysbiotic and superinfected human cancers.

## Results

### Establishment of an in vitro system that mimics the cross talk of carcinoma cells and neutrophils in the presence of bacterial stimulation

**Fig. 1:**
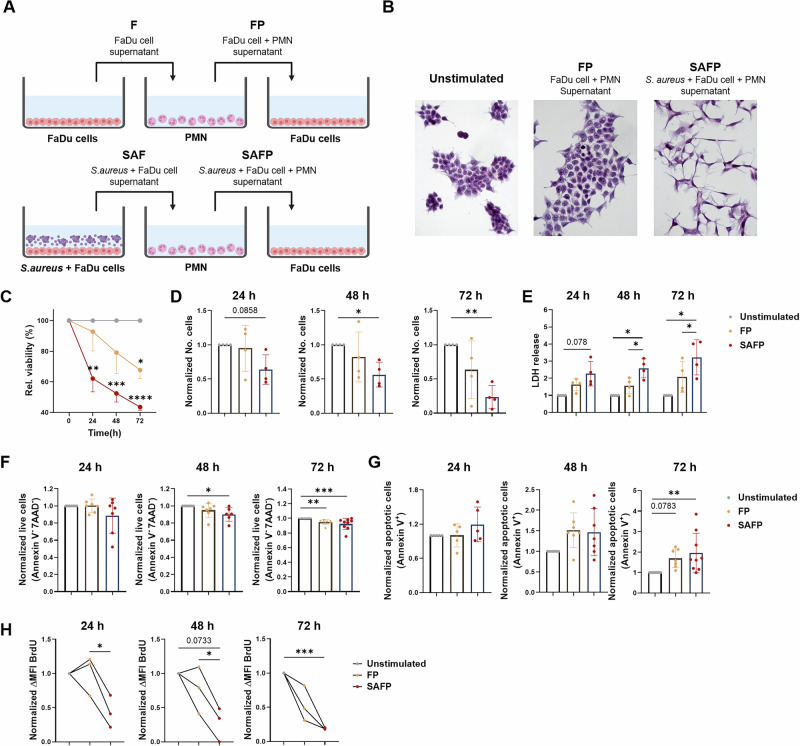
Bacterial stimulation triggers antitumorigenic tumor cell–neutrophil cross-talk. The description and nomenclature of the supernatants can be found in Table S3. **A** Upper part: For F production, FaDu cells were cultured for 24 h. For FP production, PMNs were stimulated with FaDu supernatant (F) for 20 h. Lower part: For SAF production, FaDu tumor cells were first ‘primed/stimulated’ with *Staphylococcus aureus* for 12 h; the supernatant was harvested, centrifuged and filtrated to remove bacteria. For SAFP production, PMNs were stimulated with SAF for 20 h. Scheme was prepared using a BioRender. **B** Hematoxylin staining was performed on FaDu cells that were stimulated with the indicated supernatants for 72 h. **C**–**G** Supernatants as indicated in **A** were used to stimulate tumor cells. An MTT assay **C**, a Casy hemocytometer total cell count **D**, LDH release **E**, and an AnnexinV/7AAD  flow cytometry assay **F**, **G** were used to determine cell counts and cell death. **H** Tumor responder cells were exposed to the indicated supernatants, and proliferation was measured via BrdU. For all the experiments, the data were normalized to unstimulated cells as a baseline. Statistical analysis was performed via the nonparametric Friedman test, followed by Dunn’s multiple comparisons test, and parametric one-way or two-way ANOVA, both followed by Tukey’s multiple comparisons test: *p < 0.05, **p < 0.01, ****p ≤ 0.0001. In **C**, the data are displayed as the means ± SDs of 4 independent experiments (*n* = 4). In **D**–**H**, the data are displayed as individual values of 3-9 independent experiments plus the mean ± SD (*n* = 3–9)

To model the cross talk of tumor cells and neutrophils in the presence of bacterial stimulation, we designed an in vitro system that allows for the controlled and sequential activation of tumor cells and neutrophils (Fig. 1A).

As a first model system, we used *Staphylococcus aureus* isolates to probe their effects on tumor–neutrophil cross talk. *S. aureus* colonizes approximately 20% of SCCHN patients, and methicillin-resistant *S. aureus* (MRSA) complicates the course of several cancers [33], as it is associated with surgical site infection and tumor superinfection. In line with previously published data [41], hypopharyngeal tumor cells (FaDu cells), which were cultured in the supernatant of PMNs that were previously exposed to the supernatant of FaDu cells (without bacterial stimulation), already exhibited a slight shift to mesenchymal morphology in responder tumor cells (upper panel in Fig. 1A, designated FP in Fig. 1B). However, full mesenchymal morphology was obtained only in the presence of additional stimulation of the FaDu “inducer” tumor cells with *S. aureus* (lower panel in Fig. 1A, designated SAFP in Fig. 1B).

Next, we tested whether the induction of this mesenchymal morphotype could be replicated with other bacterial strains and with different cancer cell lines. As shown in Fig. S1A (abbreviations for the supernatants are explained in Table S3), we identified two groups of bacteria-tumor-PMN supernatants: Group 1 caused morphological changes in tumor cells upon stimulation (categorized as “responders”), whereas Group 2 did not induce such changes (categorized as “nonresponders”). Visual microscopic inspection of the stimulated tumor cell cultures suggested reduced cell counts under SAFP conditions in “responders”. To confirm and quantify this observation, we performed different assays measuring cell count, cell death and proliferation (Fig. 1C–H). In conjunction, these experiments revealed a reduced cell count (Fig. 1C, D, MTT, cell counter), which was jointly caused by the induction of cell death (Fig. 1E–G, LDH release, AnnexinV/7AAD assay) and by the reduced proliferation of bacterially stimulated cells (Fig. 1H, BrdU incorporation). These initial experiments revealed that activated neutrophils secrete soluble factors that have antitumorigenic activity, directly damage tumor target cells and limit tumor cell proliferation.

**Fig. 2:**
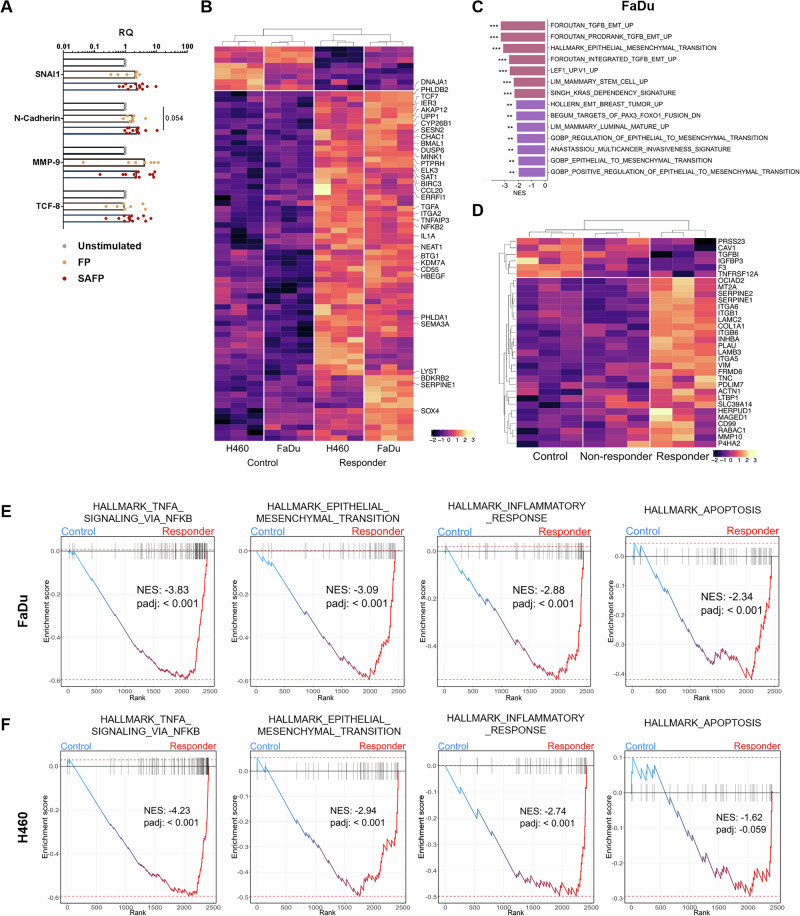
Neutrophil cytotoxicity-resistant mesenchymal tumor cells display a prometastatic and EMT-like transcriptional program. A description of the supernatants can be found in Table S3. **A** qPCR was performed on FaDu cells after 6 h of stimulation with the supernatants. The results are normalized to β-actin and untreated cells as a reference sample (RQ = 1); *n* = 7, *n* indicates the number of independent experiments. Supernatants are designated as follows: Control = untreated FaDu/H460 tumor cells, responder = cells treated with SAFP/SAHP supernatant. **B** Heatmap depicting DEGs between the control and responder samples. The genes shown correspond to all genes with a *p*-adjusted value < 0.05 from the list of selected genes shown in Table S4. The labeled genes correspond to those whose *p*-adjusted values are < 0.001. The complete set of genes is shown in Fig. S3A. **C** Gene set enrichment analysis (GSEA) was performed on the differentially expressed genes between the control and responder samples from FaDu. Gene sets containing the word ‘EMT’ were selected for the analysis. All gene sets enriched with a *p* value < 0.01 were enriched in responder samples. There were no pathways enriched in the control samples. **D** Heatmap showing differentially expressed genes between control and responder FaDu samples with a *p* value < 0.1. The genes corresponding to p-EMT genes are from [Ref. 42]. The corresponding GSEA plot is shown in Fig. S3D. GSEA plots are shown for selected pathways enriched with differentially expressed genes (Table S4) between control and responder FaDu **E** and H460 **F** samples. NES and padj (*p*-adjusted) values for the specific pathway are indicated on each plot. The corresponding heatmaps are shown in Fig. S4 (FaDu) and S5 (H460)

In the next series of experiments, we explored the cell biological changes that occurred in those tumor cells that survived exposure to activated neutrophil SAFP supernatant. We found that the morphological changes in surviving tumor cells in the “responder group” were accompanied by slight upregulation of selected mesenchymal markers (Fig. 2A). More importantly, broad transcriptional profiling by bulk RNA-seq and UMAP analysis confirmed the segregation of responders and nonresponders for two independent cell lines (Fig S2A) with several metastasis-related genes differentially expressed (Fig. S2B). For further transcriptome analysis, we selected 384 genes linked to metastasis on the basis of the available literature (genes listed in Table S4). Analysis of these genes separated responder cells from control and nonresponder cells, suggesting a prometastatic transcriptomic profile of the mesenchymal morphotype (Fig. 2B, Fig. S2C). To further explore differences between control and responder cells, we performed GSEA with a focus on EMT-related gene sets. For both FaDu and H460 cells, all tested EMT gene sets were enriched in responder cells (Fig. 2C, Fig. S3B). Puram et al. [42] profiled primary and metastatic ecosystems in HNC and established a p-EMT program in a subset of primary tumor cells as an independent predictor of nodal metastasis. We tested this patient-derived signature in our in vitro model and found that it was strongly enriched in responder samples in both cell line models (Fig. 2D, Fig. S3C–E). In particular, members of the serine protease inhibitor E (SERPINE) family, integrins and several collagen genes, all of which were previously connected to EMT, were overexpressed in responder cells. Consequently, the hallmark EMT gene set was among the most strongly enriched gene sets in responder cells compared with control cells (Fig. 2E, F, Fig S4, Fig. S5). Interestingly, p-EMT-like responder cells also overexpressed several antiapoptotic genes (e.g., MCL1, BCL2L1, BIRC3, IER3, and XIAP), which is in line with their resistance to neutrophil cytotoxicity and their induced p-EMT transcriptomic signature (Fig. S4D). In summary, these data show that our in vitro cross-talk model generates tumor cells featuring several elements of previously reported EMT programs.

### Validation of the in vitro system in human patient samples and identification of bacterially colonized intratumoral niches

**Fig. 3:**
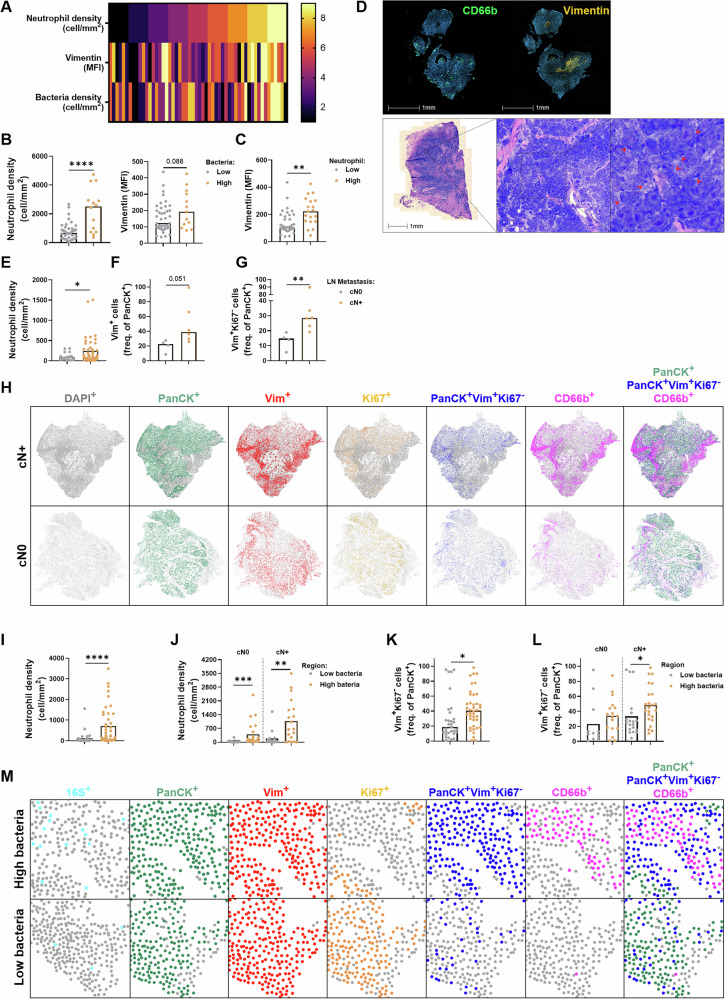
High intratumoral densities of neutrophils and bacteria are associated with a mesenchymal tumor phenotype and nodal metastasis. Tumor tissues from 54 SCCHN patients (cohort 1, Table S1) were stained with fluorescent antibodies against CD66b (neutrophils) and vimentin (mesenchymal marker). Bacteria were visualized via Pappenheim staining. The quantification of cell density and median fluorescence intensity (MFI) was performed on tumor island areas, which are displayed as color codes **A** or grouped into regions of high and low bacterial **B** or high and low neutrophil **C** density. **D** A representative IF image of CD66b and vimentin staining of total tumor tissue is depicted in the upper panel; an example of Pappenheim staining used to detect bacterial colonization (red arrows) is shown in the lower panel. **E** Tumor tissue from 65 HNC patients (cohort 2, Table S2) was stained for intratumoural neutrophils and grouped according to nodal metastasis status (gray for cN0 and yellow for cN+ ). **F**–**M** Patients without lymph node metastasis (cN0, 4 patients) and with positive node status (cN+, 6 patients) were selected for further analysis. Consecutive slides were prepared and stained with an RNAscope for the 16S ribosomal unit to detect bacterial RNA and with fluorescent antibodies directed against CD66b (neutrophils), pancytokeratin (PanCK, tumor cells), Ki67 (proliferative cells), and vimentin (mesenchymal marker). **F, G** Quantification of the frequencies of the indicated markers per patient is shown according to metastasis status. **H** Representative spatial plots showing the spatial distribution of total cells (DAPI^+^, gray), tumor cells (PanCK^+^, green), vimentin-expressing cells (Vim^+^, red), proliferative cells (Ki67^+^, orange), nonproliferative tumor cells expressing vimentin (PanCK^+^Vim^+^Ki67^-^, blue) and neutrophils (CD66b^+^, pink). Spatial plots were generated via HALO AI®. **I** Quantification of neutrophil density (CD66b^+^) according to bacterial (16S^+^) enrichment in SCCHN tissue samples. For this analysis, 5 patients were selected, and regions with high or low bacterial loads were analyzed (51 high-bacteria ROIs and 49 low-bacteria ROIs). **J**–**L** Quantification of the indicated markers in regions with high or low bacterial loads (41 high bacterial ROIs and 31 low bacterial ROIs) from selected patients with (cN+, 6 patients) and without lymphatic metastasis (cN0, 4 patients). **M** Representative spatial plots showing the spatial distribution of bacterial RNA (16S^+^), tumor cells (PanCK^+^, green), vimentin-expressing cells (Vim^+^, red), proliferative cells (Ki67^+^, orange), nonproliferative tumor cells expressing vimentin (PanCK^+^Vim^+^Ki67^-^, blue) and neutrophils (CD66b^+^, pink). Spatial plots were generated via HALO AI®. Statistical analysis was performed via the nonparametric Kruskal‒Wallis test, followed by Dunn’s multiple comparisons test or the *t* test/Mann‒Whitney test: *p < 0.05, **p < 0.01, ****p ≤ 0.0001. The data are displayed as the means or medians, with symbols representing patients **B**–**G** or intratumoral regions **I**–**L**

To explore the relevance of our in vitro interaction model for human disease, we quantified bacterial infiltration, the mesenchymal marker vimentin and neutrophil infiltration in 54 patients with SCCHN (Fig. 3A–D). We observed that bacterially colonized cancers had higher PMN counts and increased expression of vimentin in tumor cell areas (Fig. 3B). In line with the in vitro data, higher PMN density was also associated with stronger expression of the mesenchymal marker vimentin (Fig. 3C, D) in this patient analysis.

The presence of p-EMT cells in primary tumors has been associated with nodal metastasis in SCCHN [42]. Therefore, we assembled a second cohort of 65 SCCHN patients from whom enough research material was available for further tissue analysis (Table S2) and explored the potential association of cervical nodal metastasis with intratumor neutrophil density. As shown in Fig. 3E, patients with clinically apparent cervical lymph metastasis had a greater intratumoral abundance of neutrophils. From this cohort, we selected ten patients for an in-depth analysis of the tumor microenvironment, including spatial analysis of intratumoral heterogeneity (Fig. 3F–M). In agreement with previous findings, patients with positive nodal status had increased expression of vimentin in primary tumor cells (Fig. 3F), and these tumor cells were characterized by reduced expression of Ki67, suggesting a mesenchymal and potentially drug-resistant state in the tumor cells [43]. Spatial analysis of larger tumor areas confirmed the presence of intratumoral regions characterized by high expression of vimentin in tumor cells and strong infiltration by CD66b^+^ tumor-associated neutrophils, especially in patients with positive nodal status (upper panel, Fig. 3H).

To further assess the influence of bacterial colonization, we investigated whether intratumoral regions strongly colonized by bacteria differ from less colonized regions. While already detectable in the “per patient-tumor” analysis (Fig. 3A–C), we found that the relationships among bacterial colonization, neutrophil density and the mesenchymal tumor cell state were considerably more pronounced when intratumoral regions or niches were explored via spatial analysis (Fig. 3I–M). There was a significantly greater density of TANs along with increased frequencies of mesenchymal (Vim^+^Ki67^-^) carcinoma cells in intratumoral niches that were strongly colonized by bacteria. Notably, this difference was detected in tumor regions from patients with negative nodal status but was more pronounced in patients with positive lymph node metastasis (Fig. 3J–M). In summary, these data suggest a functional interplay of bacteria, neutrophils and tumor cells in HNC patients, promoting a mesenchymal and potentially metastatic tumor cell morphotype.

### Functional validation of the invasive and metastatic tumor cell phenotypes

**Fig. 4:**
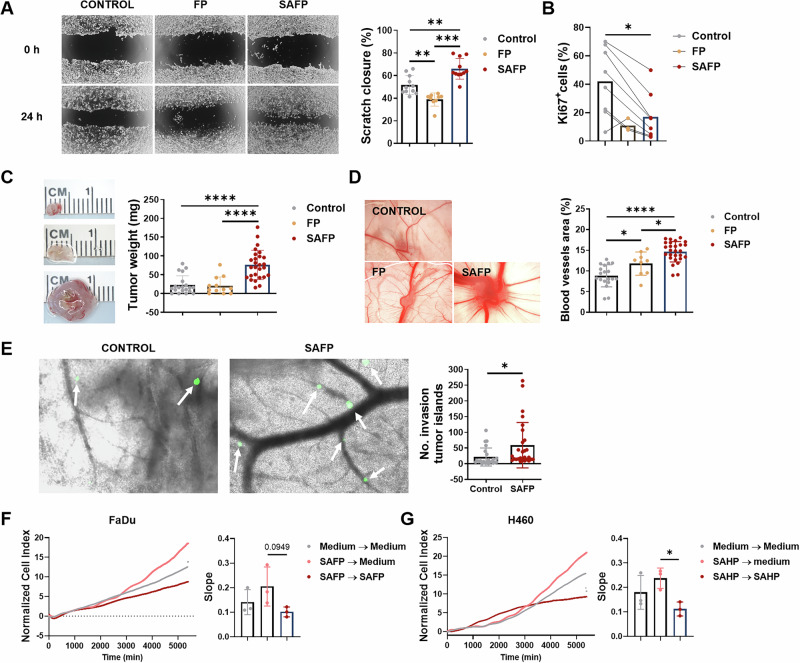
Bacterial stimulation enhances the migratory and invasive abilities of tumor cells. A description of the supernatants can be found in Table S3. **A** Control and stimulated FaDu cells were grown until confluence. Closure of a scratch was recorded after 24 h. The closure area was quantified with ImageJ; *n* = 11. **B** Ki67 expression in stimulated and unstimulated tumor cells was determined via immunofluorescence staining (control/SAFP, *n* = 8; FP, *n* = 3). **C**–**E** *In ovo* CAM assays: Stimulated tumor cells were inoculated onto the upper CAM (control *n* = 20, FP *n* = 12, SAFP *n* = 27). Macromorphology, tumor weight **C**, and angiogenesis **D** were recorded on day 3 postinoculation. **E** GFP-labeled, stimulated FaDu cells were injected into the main CAM vein of a developing chicken embryo. After 5 days, fluorescent tumor cells (marked by white arrows) in the punches obtained from the lower CAM were counted (control *n* = 23, SAFP *n* = 27). **F**, **G** Tumor cells were cultured in control medium or stimulated with either SAFP or SAHP for 72 h. The supernatant was removed, and the cells were reseeded as indicated. Increased cell growth after removal of the “inducer” supernatant was observed (*n* = 3). **A, B, F**, **G** *n* indicates the number of independent experiments, **C**–**E** *n* indicates the number of eggs used in the experiment. **A**–**F** Statistical analysis was performed with an unpaired *t* test **A, B, E, F** and one-way ANOVA with Tukey’s multiple comparisons test **C, D**: *p < 0.05, ** *p* < 0.01, *** *p* < 0.001, **** *p* ≤ 0.0001. The data are displayed as the means ± SDs

On the basis of these observations in patients, we returned to the in vitro system to explore and functionally validate the metastatic and invasive features of the transcriptional p-EMT cell state. Scratch assays revealed that the mesenchymal morphotype was indeed associated with increased migration (Fig. 4A, Fig. S1B) and reduced expression of the proliferation-associated protein Ki67 (Fig. 4B, Fig. S1C).

In the *in ovo* chorioallantoic membrane (CAM) model of fertilized chicken eggs, mesenchymal SAFP cells formed significantly larger tumors on the upper CAM than FP-stimulated and unstimulated control cells did (Fig. 4C) and significantly increased angiogenesis (Fig. 4D).

To investigate distant metastasis via the bloodstream, we injected fluorescently labeled tumor cells into the main CAM vein of the developing embryo, and after 5 days, we quantified tumor cells by counting fluorescent cells in the distant lower CAM. While differences between tumor cell populations were less pronounced than effects on upper CAM tumor development, we detected substantially greater numbers of tumor cells under SAFP conditions in some experimental eggs (Fig. 4E). Interestingly, and in line with their enhanced aggressiveness in the CAM model, monitoring of cell growth in the xCELLigence system revealed accelerated growth of the mesenchymal morphotype after removal of the induced SAFP or SAHP supernatant (Fig. 4F, G, respectively). Notably, control experiments revealed that functional changes in tumor cells were not induced in tumor cells that were directly stimulated with bacteria or exposed to supernatants from bacterially stimulated tumor cells, thus mimicking autocrine stimulation in the absence of the PMN (data not shown).

### Characterization of PMN activation induced by bacterially stimulated tumor cells

**Fig. 5:**
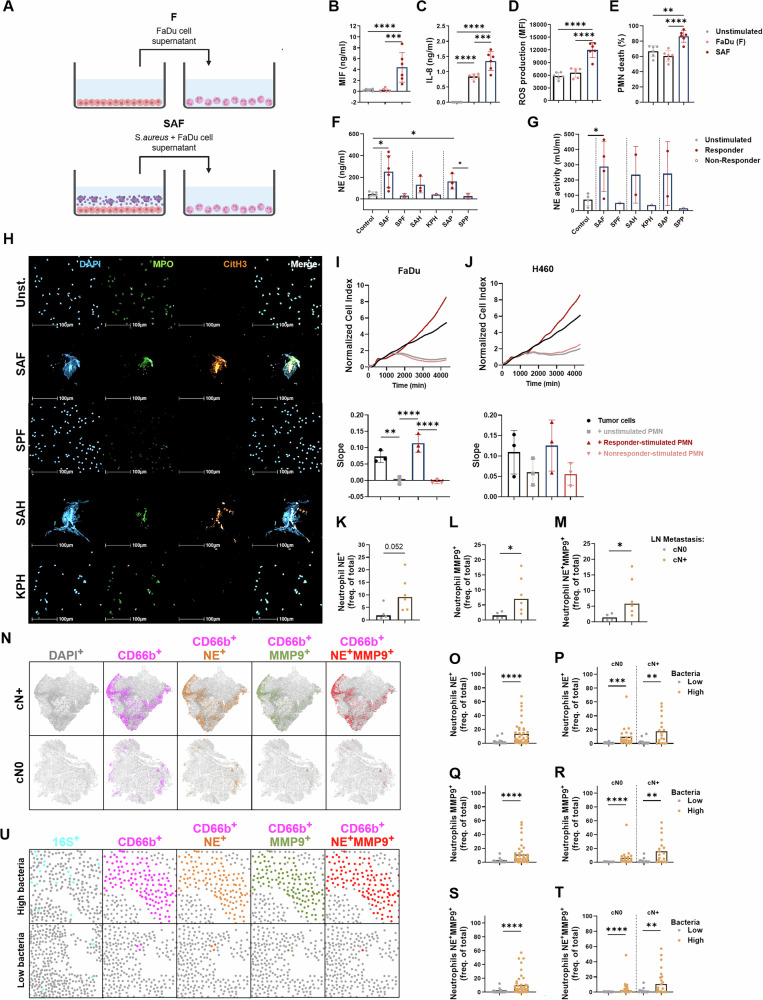
Bacterial stimulation of tumor cells induces PMN activation. A description of the supernatants can be found in Table S3. **A** Exemplary scheme of PMN stimulation: Upper part: Control PMNs (unstimulated) were treated with the supernatant of untreated FaDu cells (FaDu (F)); lower part: PMNs were treated with supernatant from bacteria-stimulated tumor cells (SAFs). Supernatants from 20 h stimulated or unstimulated PMNs were harvested, and the concentrations of MIF **B** and IL-8 **C** were determined via ELISA, *n* = 6. **D** PMNs were stimulated for 3 h, and ROS production was determined via 123Dihydrorhodamine and flow cytometry, *n* = 6. **E** PMNs were stimulated for 24 h, and the number of dead cells was determined via AnnexinV/7AAD staining and flow cytometry, *n* = 6. Supernatants from PMNs stimulated for 20 h under the indicated conditions were harvested, and the concentration **F** and activity **G** of NE were determined, *n* = 1–6. **H** NET formation was analyzed in PMNs stimulated for 30 min with different supernatants. The release of myeloperoxidase (MPO, green) and citrullinated histone 3 (CitH3, orange) was detected by immunofluorescence to confirm NET formation, *n* = 3. **I**, **J** PMNs were stimulated with the designated supernatants for 30 min and subsequently added to tumor cells on an E-plate. The proliferation of tumor cells was monitored with xCELLigence for 72 h, *n* = 3. Tumor tissues from ten SCCHN patients (also shown in Fig. 3F–H) were stained for CD66b (neutrophils), neutrophil elastase (NE) and matrix metalloproteinase-9 (MMP-9). Patients were grouped according to their nodal metastasis status (gray for cN0 and yellow for cN+  ); the frequencies of neutrophils expressing NE (CD66b^+^NE^+^) **K**, MMP-9 (CD66b^+^MMP-9^+^) **L**, and both markers (CD66b^+^NE^+^MMP-9^+^) **M** in the tumor tissue were quantified. **N** Representative spatial plots showing the distribution of total cells (DAPI^+^, gray), neutrophils (CD66b^+^, pink), and neutrophils expressing NE (CD66b^+^NE^+^, orange), MMP-9 (CD66b^+^MMP-9^+^, green), or both markers (CD66b^+^NE^+^MMP-9^+^, red). **O**–**T** Frequencies (as a percentage of total DAPI^+^ cells) of neutrophils expressing NE (CD66b^+^NE^+^), MMP-9 (CD66b^+^MMP-9^+^), or both markers (CD66b^+^ NE^+^MMP-9^+^) in SCCHN tissue are shown according to bacterial (16S^+^) enrichment. For this analysis, regions showing high and low bacterial colonization were analyzed (41 high bacterial ROIs and 31 low bacterial ROIs). **U** Representative spatial plots showing the spatial distribution of bacterial RNA (16S^+^), neutrophils (CD66b^+^, pink), neutrophils expressing NE (CD66b^+^NE^+^, orange), MMP-9 (CD66b^+^MMP-9^+^, green), and both markers (CD66b^+^ NE^+^MMP-9^+^, red) in regions with high or low bacterial loads. **B**–**E, H** *n* indicates the number of independent PMN donors tested. **F**, **G** *n* indicates the number of independent technical replicates. **I, J** *n* indicates the number of independent experiments and PMN donors. **K**–**M** Each symbol represents one patient. **O**–**T** Each symbol represents one unique intratumoral region. All the statistical analyses were performed via unpaired *t* tests: *p < 0.05, ** *p* < 0.01, *** *p* < 0,001, **** *p* ≤ 0.0001. The data are displayed as the means ± SDs. Spatial plots were generated via HALO AI®

To mechanistically decipher the role of neutrophils in our model, we first tested whether bacterially stimulated tumor cells activate the PMN (Fig. 5A). Indeed, bacterially stimulated tumor cells (SAFs) were superior to unstimulated tumor cells (F=FaDu) in inducing cytokine release (Fig. 5B, C), ROS production (Fig. 5D), cell death (Fig. 5E), NE release and activity (Fig. 5F, G) and the formation of NETs (Fig. 5H). Interestingly, NE and NETs were induced by “responder” (SAF, SAH) but not by “nonresponder” supernatants (SPF, KPH). In addition, PMN exposure to “nonresponder” supernatants reduced tumor cell growth, whereas PMN stimulation with “responder” supernatants accelerated growth in two different cell line systems of PMN-tumor coculture (Fig. 5I, J). It has been previously reported that NETs and NET-associated functional molecules, such as NE and MMP-9, can induce migration and invasion in certain types of cancer [44–47]. Since NET formation was a prominent feature of “responder” supernatants in our system, we tested whether NE and MMP-9, two main components of NETs, could drive the observed tumor cell changes. Both Sivelestat, an NE inhibitor, and GM6001, an MMP-9 inhibitor, affected major features of the metastatic tumor cell phenotype (Fig. S6, Fig. S7). Blockade of NE during the neutrophil-mediated induction phase modulated EpCAM expression, resulting in recovery of tumor cell proliferation, reduced tumor cell migration and reduced CAM tumor growth and angiogenesis (Fig. S6). Similarly, the application of GM6001 during the induction phase partially reduced the induction of MMP-9 in responder tumor cells, scratch closure, upper CAM tumor weight and angiogenesis (Fig. S7). Capitalizing on material and data from patients with oropharyngeal and oral cavity carcinomas in our second patient cohort (Table S2), we found that the abundance of neutrophils expressing NE and MMP-9, alone or in combination, was greater in patients with clinically positive cervical lymph node status (Fig. 5K‒M). Spatial analysis allowed us to map the expression of NE and MMP-9 (Fig. 5N) to intratumoral niches previously identified to harbor vimentin^+^ carcinoma cells (Fig. 3H; note that the same tumors are shown in Fig. 3H and Fig. 5N). We next focused on intratumoral regions that we defined as “bacteria high” or bacteria low” according to the bacterial 16S RNA signal density. In line with the in vitro data, which revealed the induction of NE and MMP-9 in neutrophils by indirect bacterial stimulation, the relative abundance of neutrophils expressing NE and MMP-9 was greater in regions strongly colonized by bacteria, with the highest values reached in patients with positive node status (Fig. 5O–U).

These data establish two neutrophil granule proteins, i.e., NE and MMP-9, as the molecular links between bacterial stimulation, intratumoral neutrophil activation and tumor phenotype switching toward a prometastatic state.

### Bidirectional cross-talk between cancer cells and tumor-associated neutrophils triggers innate immune escape

**Fig. 6:**
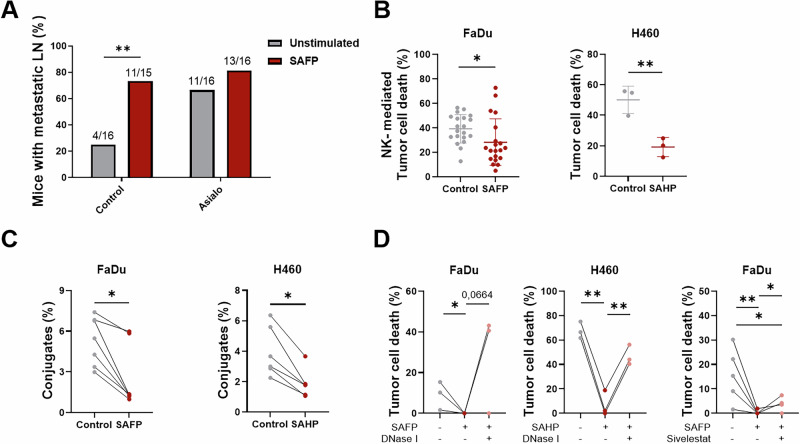
SAFP-stimulated mesenchymal tumor morphotypes are resistant to NK cytotoxicity. A description of the supernatants can be found in Table S3. **A** Unstimulated tumor cells (16 mice total) or tumor cells stimulated with SAFP supernatant (15 mice total) were injected into the floor of the mouth of nude mice. After 20 days, the mice were sacrificed, and the percentage of mice with lymph node metastases was determined. The mice were treated either with normal rabbit serum (control) or with an anti-asialo GM1 antibody for the depletion of NK cells. **B** Tumor cells were stimulated with supernatants for 72 h, IL-2-stimulated NK cells were added for 24 h, and NK cell-mediated tumor cell death was determined via an AnnexinV/7AAD flow cytometry assay (FaDu: control, *n* = 20; SAFP, *n* = 22; H460: control; SAHP, *n* = 3). **C** Tumor cells were stimulated with supernatants for 72 h, and NK cells were stimulated with IL-2 for 48 h. Afterwards, both cell types were fluorescently labeled and placed together to form conjugates, which were tracked via flow cytometry (FaDu *n* = 7, H460 *n* = 6). **D** Tumor cells were stimulated with supernatants for 72 h (with the addition of DNase I or Sivelestat during supernatant production), IL-2-stimulated NK cells were added for 24 h, and tumor cell killing was determined via an AnnexinV/7AAD flow cytometry assay (DNase I experiment, *n* = 3; Sivelestat experiment, *n* = 5). **A** Numbers of metastatic mice and total mice per group are indicated. **B**–**D** *n* indicates the number of experiments, each with an independent NK cell donor. Statistical analysis for all experiments was performed with an unpaired *t* test: *p < 0.05, ** *p* < 0.01. The data are displayed as the means ± SDs

In the final series of experiments, we aimed to functionally validate the metastatic phenotype in vivo. To this end, we utilized a previously established murine model of orthotopic HNC tumor cell inoculation [17]. When we injected SAFP-stimulated tumor cells into the floor-of-mouth of nude mice, the regular low nodal metastasis rate (approximately 20%) increased to over 70% (Fig. 6A, left two bars). NK cells are known to be important players in the control of metastatic dissemination [48]. Since nude mice lack T cells but retain fully functional NK cells, our animal model offered an opportunity to investigate potential escape from innate immune control by SAFP-stimulated tumor cells. Interestingly, we observed that the difference in nodal metastasis was abrogated upon depletion of NK cells by injection of asialo-GM1 antibodies (Fig. 6A, right two bars), suggesting that metastasis formation is indeed controlled by NK cells. In vitro cytotoxicity assays revealed a decrease in the killing of SAFP-stimulated cells by human NK cells (Fig. 6B), which corroborated these findings. Since the regulation of activating (MICA) and inhibitory (MHC-I) NK ligands on tumor cells was not different between control and mesenchymal tumor cells (data not shown), we investigated the physical cell‒cell contact interaction and conjugation between NK cells and their targets via an in vitro conjugation assay. Indeed, as shown in two independent cell lines, mesenchymal metastatic tumor cells formed significantly fewer conjugates with NK cells (Fig. 6C). Finally, we tested whether PMN NETs may form a physical barrier between tumor cells and NK cells to limit cell‒cell contact and thus NK cytolytic activity. Destruction of NETs by both DNAse I and NE inhibition reversed the resistance of metastatic tumor cells to NK cells (Fig. 6D). These data show that bacterial stimulation of tumor cells leads to indirect PMN activation and NET formation, which results in a physical barrier between tumor cells and NK cells that is equivalent to innate immune escape and hinders immunologic control of metastatic p-EMT cells.

## Discussion

Studies in gastrointestinal cancers have elucidated the important role of the gut microbiome in tumor progression and response to therapy [35, 36]. Similarly, the oropharyngeal and respiratory tracts are populated by commensal microbes [37], and changes in the local microbiome composition have been shown to be associated with local tumor growth [49]. In addition to commensals, pathogenic bacteria [49] such as *S. aureus, S. pneumoniae* and *K. pneumoniae* are often associated with cancers of the upper respiratory tract. While it is not yet clear whether local infections may promote tumorigenesis or whether infections occur once the tumor has disrupted the local mucosal barrier, bacterial colonization of the tumor and its microenvironment appears to play a role in tumor progression [40]. Here, we aimed to identify the underlying mechanisms by investigating the effects of bacteria on tumor-neutrophil crosstalk.

Neutrophils respond directly to such microbial challenges and play important roles in immuno-oncology. Solid tumors, including lung cancer and head and neck cancers, are commonly infiltrated by considerable numbers of tumor-associated neutrophils [50]. In most cases, these high TAN frequencies are associated with poor patient outcomes [51]. Multiple mechanisms that could explain this phenomenon have been proposed in recent years [52]. In this context, TANs have been implicated in the promotion of local tumor progression as well as the induction of metastasis and angiogenesis. More recently, the immunosuppressive activity of TANs has attracted considerable interest. Neutrophils with T-cell-suppressive myeloid-derived suppressor cell (MDSC) activity have been identified as promoters of HNC and other types of cancer [20, 53].

Despite this progress in the field, how bacteria influence the cross-talk between tumor cells and TANs is currently unknown. In this study, we established an in vitro model of sequential cellular activation that mimics the cross-talk between TANs and tumor cells in the presence of distinct bacterial species. In this system, we found that bacterial stimulation of tumor cells led to subsequent neutrophil activation. The secretome of these activated neutrophils has direct antitumor activity, as illustrated by their tumor-cytotoxic and antiproliferative effects. These findings are in line with several reports in the literature that demonstrated direct antitumorigenic effects of stimulated neutrophils [16–19].

Interestingly, in parallel with this antitumor activity, activated neutrophils also trigger a phenotypic switch in surviving tumor cells, as illustrated by enhanced metastasis and immune (NK) escape. These mesenchymal tumor cells showed enhanced migration in vitro as well as enhanced local and metastatic tumor growth in CAM and murine orthotopic cancer models. Thus, in a system of bacterially triggered indirect neutrophil activation, we observed parallel induction of antitumor (tumor cell death, reduced proliferation) and protumor (prometastatic, innate immune escape) activity in primary human neutrophils.

This finding is intriguing, as the mechanisms governing neutrophils´ dual pro- and anti-tumor activities remain elusive, despite extensive research over the past 15 years [14, 54, 55]. Initially, it was thought that the neutrophil dichotomy primarily stems from the presence or absence of tumor microenvironmental factors that promote either protumorigenic (TGF-b) [54] or antitumorigenic (type I IFN) [56] neutrophil activity. More recently, this initial concept has been refined to reflect the existence of multiple different neutrophil phenotypes (and inducers) associated with protumor or antitumor activity [57]. Very recently, RNA-seq technology has been optimized for the analysis of neutrophils [58, 59]. This technological advance revealed remarkable transcriptional plasticity and diversity in neutrophils [24–28] comparable to what has been observed for other myeloid cell subsets. As a consequence, multiple different functional phenotypes of TANs have been reported, and in some cases, distinct protumorigenic or antitumorigenic activities could be associated with those subsets [24, 29–31]. According to current hypotheses, the induction of those transcriptional and functional states is regulated by a not yet understood intrinsic receptivity of the neutrophil population per se in conjunction with the presence of tumor microenvironmental factors that promote neutrophil diversity [15].

Our study provides an alternative scenario that likewise explains the functional neutrophil dichotomy. We show that neutrophil pro- or antitumor activity is regulated at the level of the tumor target cell itself. When we exposed the tumor cell population to conditioned medium that mimics bacteria-tumor-neutrophil cross-talk, a proportion of the tumor cells were killed. However, and importantly, the surviving tumor cells acquired a mesenchymal and prometastatic phenotype. This phenotype exhibited aggressive growth in vitro, in a CAM model and in an orthotopic preclinical murine model. According to this model, the net pro- or antitumor activity of neutrophils depends on the relative susceptibility or resistance of the target tumor cells to cytotoxic mediators, antiproliferative mechanisms and prometastatic signals.

In conjunction, our findings suggest that prometastatic bidirectional cross-talk between tumor cells and neutrophils is stimulated by bacterial superinfection. While our sequential in vitro stimulation system allows the identification of causal and mechanistic connections between bacteria, tumor cells and neutrophils, we acknowledge that in the patient TME, this interaction is multidirectional. Thus, other potential interactions, such as the antimicrobial activity of neutrophils, are not part of our analysis. However, even in view of these limitations, the human tissue data clearly revealed a correlation between the mesenchymal tumor phenotype, total intratumoral bacterial load, TAN frequency and nodal metastasis, thus reinforcing the mechanistic relevance of this bacterially enhanced protumorigenic-TAN cross-talk in patients. Via spatial analysis, we also identified certain intratumoral areas or niches that are characterized by the coabundance of neutrophils, bacteria and mesenchymal tumor cells. The presence of these areas is associated with increased nodal metastasis in patients with HNC. Finally, our study also provides some initial experimental evidence that may eventually lead to an alternative biological explanation for the functional neutrophil dichotomy in cancer patients.

## Materials and methods

### Study subjects

Polymorphonuclear neutrophils (PMNs) and natural killer (NK) cells were isolated from the peripheral blood of healthy donors and used in in vitro systems. Human malignant tissues were collected from two cohorts of patients with squamous cell carcinoma of the head and neck (SCCHN). The clinical characteristics of the patients in cohorts 1 and 2 are shown in Tables S1 and S2, respectively. All studies were approved by the local ethics committee, and informed written consent was obtained from each individual.

### Immune cell isolation

Diluted blood (1:1, v/v in phosphate-buffered saline (PBS)) was subjected to density gradient centrifugation via Pancoll separating solution density 1 (Pan-Biotech, Aidenbach, Germany). The mononuclear cell fraction was aspirated or used for NK cell isolation. The neutrophil fraction was collected in a fresh test tube. Erythrocytes were removed by sedimentation with a solution containing 1% polyvinyl alcohol and, subsequently, by lysis with 0.2% NaCl solution. Reconstitution of osmolarity was obtained by adding 1.2% NaCl. The resulting PMNs were cultured in RPMI-1640 supplemented with 10% fetal calf serum (FCS), 100 units/ml penicillin and 100 µg/ml streptomycin (hereafter designated culture medium). NK cell isolation was performed via an NK isolation kit II (MACS) according to the manufacturer’s protocol (Miltenyi BioTec, Bergisch-Gladbach, Germany). Purified NK cells were cultured in culture medium.

### Cell lines and bacteria used in the study

The human carcinoma cell lines FaDu (RRID:CVCL_1218), PCI1 (RRID:CVCL_C167) and H460 (RRID:CVCL_0459) were cultured in RPMI-1640 supplemented with 10% fetal calf serum (FCS), 100 units/ml penicillin and 100 µg/ml streptomycin. This medium was also used for all the cell lines used for the generation of supernatants (see below). The bacterial strains *Staphylococcus aureus* (ATCC25923), *Streptococcus pneumoniae* (ATCC6305), and *Klebsiella pneumoniae* (ATCC700603) were cultivated in LB media at 37 °C under shaking conditions. Human carcinoma cell lines were regularly tested for mycoplasma contamination via the Venor ® GeMClassic Mycoplasma Detection Kit (Minerva Biolabs).

### Production of conditioned supernatants

#### Production of tumor supernatant (Fig. 1A, upper part, abbreviated F)

Tumor cells were cultured in culture medium at a concentration of 2×10^6^/ml for 24 h at 37 °C and 5% CO_2_. The supernatant was collected, centrifuged at 2760xg for 10 min and stored at -80 °C.

#### Production of tumor-PMN supernatant (Fig. 1A, upper part, abbreviated FP)

PMNs were isolated from the blood of at least 3 healthy donors and stimulated with tumor supernatant (F) at a concentration of 1x10^6^/ml for 20 h at 37 °C and 5% CO_2_. Next, the supernatants were harvested, centrifuged two times at 2760xg for 15 min and filtered with a 0.2 µm filter. Afterwards, supernatants from all PMN donors were pooled and stored at -80 °C (FP).

#### Production of bacteria-tumor supernatant (Fig. 1A, lower part, abbreviated SAF)

Tumor cells were seeded at a concentration of 2×10^6^/ml in culture medium without antibiotics. Bacteria were cultured in LB medium until they reached an OD = 0.6. OD measurements were performed using a Synergy2 plate reader (BioTek). After approximately 4 h of adherence, the tumor cells were stimulated with 10^6^ bacteria/ml (MOI of 0.5) for 12 h at 37 °C and 5% CO_2_. The supernatant of the coculture (SAF) was harvested, and the remaining bacteria were removed via two steps: centrifugation at 2760xg for 15 min and filtration through a 0.2 µm filter. Additionally, to prevent further bacterial contamination, 1% penicillin/streptomycin solution was added. The collected supernatant was stored at -80 °C.

#### Production of bacteria-tumor-PMN supernatant (Fig. 1A, lower part, abbreviated SAFP)

PMNs were isolated from the blood of 3-4 healthy donors and stimulated at a concentration of 10^6^/ml with previously prepared bacteria-tumor supernatant (SAF). After 20 h of incubation at 37 °C and 5% CO_2_, the supernatants were harvested, centrifuged two times at 2760xg for 15 min and filtered with a 0.2 µm filter. Afterwards, supernatants from all the PMN donors were pooled and stored at −80 °C (SAFP). Note that we used PMNs from healthy donors for such experiments to mimic the conversion of “normal” neutrophils to tumor-associated neutrophils in the presence of tumor-derived factors.

A list of all the supernatants used in this study can be found in Table S3.

### Treatment with inhibitors

During the stimulation of PMNs with bacteria-tumor supernatant (SAF) containing 10 µM Sivelestat (Sigma‒Aldrich), 10 µM 200GM6001 (Tocris) or 0.5 U/ml DNAse I (Sigma‒Aldrich) was added. The supernatants containing the inhibitors were harvested according to the protocol described above.

### Hematoxylin staining

FaDu tumor cells were stimulated on glass coverslips in a 24-well plate for 72 h in control medium or conditioned media. The cells were fixed for 10 min with 4% PFA, washed with PBS and stained for 30 s with Shadows Hematoxylin (Thermo Fisher Scientific, Karlsruhe, Germany), followed by blueing in tap water for 10 min. Images were taken via the Zeiss Axioscope2 and AxioCamMRC with a Ph2 Plan-Neofluar 20x/0.50 objective.

### Cell counting and viability assay

FaDu cells were stimulated for 24, 48 or 72 h with control medium or conditioned media, as described above. Tumor cells were counted via an electronic cell counter (Casy Model TT; Omni Life Science) following the manufacturer’s instructions. Live and apoptotic cells were determined via an AnnexinV/7AAD staining kit (BD Bioscience) according to the manufacturer's protocol. The samples were analyzed via a BD FACSCanto II flow cytometer and BD FlowJo software.

### MTT assay

FaDu cells were cultivated in 96-well plates in control medium or conditioned media for 24, 48 and 72 h at 37 °C and 5% CO_2_. At the end of the stimulation, the supernatants were removed for LDH analysis, and the cells were incubated in 5 mg/ml 3-(4,5-dimethylthiazol-2-yl)-2,5-diphenyltetrazolium bromide (MTT) solution for 4 h at 37 °C and 5% CO_2_. Afterwards, the plates were centrifuged, the MTT medium was discarded, and the formazan crystals were dissolved in dimethyl sulfoxide (DMSO) and measured at 630 nm via a Synergy™ 2 microplate reader (Biotek).

### LDH release assay

LDH release was measured via the CytoTox 96® Non-Radioactive Cytotoxicity Assay (Promega) according to the manufacturer's protocol. Supernatants from stimulated FaDu cells were collected after 24, 48 and 72 h and mixed with CytoTox 96® Reagent (1:1 ratio). After 30 min of incubation, CytoTox 96® Stop Solution was added to each sample, and the absorbance was measured at 490 nm via a Synergy™ 2 plate reader (Biotek).

### BrdU proliferation assay

The proliferation of tumor cells stimulated with conditioned supernatants for 24, 48 and 72 h was assayed via a BrdU Staining Kit (eBioscience™, Invitrogen) according to the manufacturer's recommendations. Briefly, tumor cells were labeled with 10 µM BrdU for the last 20 h of incubation. Tumor cells were costained with the Fixable Viability dye eFluor™ 780 (eBioscience™, Invitrogen), followed by a fixation/permeabilization step and DNase I treatment. Finally, the tumor cells were costained with an antibody against BrdU conjugated with PerCP-eFluor™ 710 (eBioscience™, Invitrogen). The samples were analyzed via a BD FACSCanto II flow cytometer and BD FlowJo Software.

### RNA sequencing analysis of stimulated tumor cells

Tumor cells were stimulated with conditioned media for 8 h at 37 °C and 5% CO_2_. RNA was isolated using NucleoSpin® RNA II, (MACHEREY-NAGEL GmbH & Co. KG, Düren, Germany), according to the manufacturer's protocol. The concentration and quality of the RNA were measured with a Qubit (Invitrogen, Waltham, MA, USA) and an Agilent Bioanalyzer DNA HS (Agilent, Santa Clara, CA, USA). Library preparation was performed with a Lexogens QuantSeq 3’ mRNA-Seq Library Prep Kit FWD and sequenced on a NextSeq500 (Illumina, San Diego, CA, USA). Adapter sequences were removed with TrimGalore [60], and sequences were aligned with HISAT2 [61]. Statistical analysis was performed with R (v. 4.2.0, R Core Team (2022)). R: A language and environment for statistical computing. R Foundation for Statistical Computing, Vienna, Austria. (URL https://www.R-project.org/) with the R packages DESeq2 [62], pheatmap [63], and umap (v 0.2.8.0); Konopka T (2022). umap: https://cran.r-project.org/web/packages/umap/, fgsea [64]. The list of genes used for metastasis connectivity analysis can be found in Table S4 and Table S5. The RNA-seq data are accessible at GEO (GSE287001).

### Gene expression analysis

Tumor cells were stimulated with conditioned media for 6 h at 37 °C and 5% CO_2_. RNA was isolated using  NucleoSpin® RNA II kit (MACHEREY-NAGEL GmbH & Co. KG, Düren, Germany). Up to 1 µg of RNA was used for reverse transcription according to the manufacturer's protocol (SuperScript™ II Reverse Transcriptase, Thermo Fisher Scientific). Real-time polymerase chain reaction (RT‒PCR) was performed on cDNA via Luna® Universal qPCR Master Mix (New England Biolabs) and the StepOnePlus Real-Time PCR System with StepOne software. All samples were standardized to untreated cells and the housekeeping gene (β-actin) via the 2^ΔΔCt^ method. The sequences of all the oligonucleotides and the corresponding annealing temperatures are indicated in Table S6.

### Immunofluorescence staining

#### IF staining of tumor cells

For immunofluorescence staining, tumor cells were seeded on coverslips for stimulation with conditioned media. Coverslips were fixed and permeabilized with BD Cytofix/Cytoperm for 30 min. Samples were stained with primary antibodies overnight at 4 °C followed by incubation with secondary antibodies for 45 min at RT. For visualization of the nuclei, 0.14 µg/ml DAPI (BioLegend) was used. The samples were mounted with Fluoromount–G Mounting medium (Thermo Fisher Scientific) and analyzed with a Zeiss Axioscope 2 and Zeiss AxioObserver. Z1 microscope. The quantification of Ki67-positive tumor cells was performed via ImageJ software. The antibodies used are listed in Table S7.

#### IF staining of tumor tissue

Tumor biopsies from SCCHN patients were frozen in Tissue-Tek O.C.T. Compound (Sakura Finetek), and 5 µm thick sections were prepared. The tissue sections were fixed and permeabilized with BD Cytofix/Cytoperm for 15 min. The samples were then stained with primary antibodies overnight at 4 °C, followed by incubation with secondary antibodies for 45 min at RT. Then, 0.14 µg/ml DAPI (BioLegend) was used to visualize the nuclei. The samples were mounted with Fluoromount–G mounting medium and analyzed with an AxioScan Z1 slide scanner. Spatial analysis was performed via either Definiens Tissue Studio™ or HALO®, a Highplex FL module (Indica Labs). Spatial plots showing marker distributions were created via the HALO® spatial analysis module. The antibodies used are listed in Table S7.

### Pappheim staining of tumor tissue sections

Bacteria in the tumor sections were visualized via Pappenheim staining. In brief, the slides were stained for 2 min with May-Grünwald solution (Merck AG, Darmstadt, Germany), washed in distilled water, counterstained for 10 min with Giemsa solution (Merck AG, Darmstadt, Germany) and mounted with a Roti Histo Kit II (Carl Roth, Kalrsruhe, Germany). Bacteria were quantified by manual counting via a Multiview microscopy system.

### RNAscope-FISH

The bacterial 16S ribosomal RNA (16S-rRNA) distribution in SCCHN tissue was assayed via multiplex fluorescent RNAscope-FISH technology (Advanced Cell Diagnostics, Newark, USA) according to the manufacturer’s instructions. Briefly, previously prepared fresh-frozen 5 µm thick tumor sections were thawed and fixed with 4% PFA for 15 min. The sections were dehydrated via immersion in 50%, 70% and 100% ethanol solutions. Next, the samples were treated with hydrogen peroxide for 10 min, followed by RNAscope Protease IV treatment for 20 min at RT. The samples were hybridized with an Eubacteria probe (RNAscope™ Probe-EB-16S-rRNA-C3, Advanced Cell Diagnostics) for 2 h at 40 °C in a HybEZ™ II hybridization oven. Following amplification and extensive washing, the 16S signal was stained with TSA Vivid Fluorophore 520 (1:3000) for 30 min at 40 °C in a HybEZ™ II oven. Finally, the samples were incubated with DAPI (BioLegend) for 5 min, after that the tissue was mounted with ProLong™ Gold Antifade Mountant solution (Thermo Fisher Scientific). The slides were scanned via a Zeiss AxioScan Z1 slide scanner.

### Scratch assay

Tumor cells were seeded and stimulated with the corresponding supernatants in 12-well plates for 24 h. The scratch was inflicted with a pipette tip, and the cells were cultured for an additional 24 h. Images were taken at two time points, i.e., 0 and 24 h. Quantification of the scratch-closure ability was performed via ImageJ software.

### Chicken chorioallantoic membrane (CAM) – *in ovo* invasion assay

The fertilized eggs were incubated at 38 °C for 10–12 days. Subsequently, stimulated FaDu tumor cells were inoculated (1.5x10^5^ cells) onto the chorioallantoic membrane (CAM), or stimulated FaDu-eGFP cells (7.5*10^4^ cells) were injected into a CAM vein as described previously^65^. Three days after inoculation, the eggs were opened, and samples from the upper CAM and tumor were collected. Five days after injection, lower CAM samples were collected. The tumor size was measured. For angiogenesis and invasion studies, images of the lower and upper CAM were collected, and the analysis was performed via ImageJ software.

### Real-time proliferation assay

After stimulation with conditioned media for 72 h, the tumor cells were harvested, counted and resuspended in either medium or conditioned supernatants. These suspensions were then seeded onto x-CELLigence E-plates (FaDu: 8x10^3^ cells, H460: 1x10^4^ cells per well). Tumor cell proliferation was monitored over the next 90 h via an xCELLigence RTCA instrument (Agilent).

### ROS assay

PMNs were cultured at 1x10^6^ cells/ml in stimulating supernatants for 3 h at 37 °C and 5% CO_2_. Afterwards, 123DiRhodamine was added at a final concentration of 2.5 µg/mL. The samples were immediately analyzed on a BD FACSCanto II flow cytometer via BD FlowJo Software.

### Cytokine detection

Supernatants from PMNs stimulated with conditioned media were collected after 20 h. ELISAs for the detection and quantification of human MIF, human IL-8 and human NE were performed according to the manufacturer’s protocol (Bio-Techne GmbH, Wiesbaden-Nordenstadt, Germany).

### NE activity assay

NE activity assays were performed via a Neutrophil Elastase Activity Assay Kit according to the manufacturer’s protocol (Cayman Chemical, Michigan, USA).

### NET formation assay

NET formation assay was performed on poly-D-lysine-coated coverslips in 24-well plates. PMNs were cultured in the respective control medium or conditioned supernatant and incubated for 30 min at 37 °C and 5% CO_2_. The cells were fixed by adding 16% PFA for 15 min, followed by a permeabilization step with 0.5% Triton X-100. Afterwards, the samples were blocked with blocking buffer (3% cold water fish gelatin, 1% BSA, 0.5% Tween-20 in PBS) for 20 min at RT. To visualize NET formation, the slides were stained with unconjugated antibodies targeting myeloperoxidase (MPO; mouse anti-human MPO; Bio-Rad) and citrullinated histone H3 (CitH3; rabbit anti-human histone H3; Abcam) for 1 h at RT. Next, the samples were stained with Alexa Fluor™ 480-conjugated donkey anti-mouse (Thermo Fisher Scientific, Molecular Probes) and Alexa Fluor™ 546-conjugated donkey anti-rabbit (Thermo Fisher Scientific, Invitrogen) secondary antibodies for 45 min at RT. The antibodies used are listed in Table S7. Finally, DAPI (BioLegend) was added for 30 min at RT in the dark. The samples were mounted with Fluoromount–G mounting medium and analyzed with a Zeiss Axioscope 2 and Zeiss AxioObserver Z1.

### PMN-tumor cell killing assay

Tumor cells were seeded onto x-CELLigence E-plates (FaDu: 8x10^3^ cells, H460: 1x10^4^ cells per well) and incubated for 5 h for initial adherence. PMNs were stimulated with medium or conditioned media for 30 min. Stimulated PMNs were then added to tumor cells (5:1 ratio), and their impact on tumor cell survival was monitored for 72 h.

### Animals

Male, 6–10-week-old NMRI-nude mice were challenged with stimulated and unstimulated FaDu cells on the floor of the mouth (*M. myelohyoideus*). For NK depletion, a rabbit anti-mouse/rat Asialo GM1 polyclonal antibody was administered to the mice via intraperitoneal injection every 5 days throughout the duration of the experiment. The first asialo-GM1 administration was performed 2 days before tumor challenge. Mice treated with normal rabbit serum were used as a control group. The mice were sacrificed between 19 and 22 days postinjection. The two pairs of superficial cervical lymph nodes were removed and embedded in Tissue-Tek O.C.T. Compound (Sakura Finetek) and cut into 5 μm sections. Every 6th section was stained with hematoxylin/eosin and analyzed to observe the presence of metastases via light microscopy. All experiments were approved by the local animal ethics committee.

### NK-mediated tumor cell killing

Tumor cells (2×10^5^/ml) were stimulated with selected supernatants in a 12-well plate for 72 h at 37 °C and 5% CO_2_. NK cells were isolated and stimulated at a concentration of 1x10^6^/ml with 200 U/ml IL-2 for 48 h at 37 °C and 5% CO_2_. Stimulated tumor cells and IL-2-activated NK cells were seeded at a 1:1 ratio in a 96-well flat bottom plate and cocultured for 24 h at 37 °C and 5% CO_2_. To assess tumor cell death, the entire culture supernatant was transferred into a new 96-well plate. Adherent tumor cells were detached and combined with the transferred supernatant. The plate was centrifuged at 460xg for 5 min, and the cells were stained with an AnnexinV/7AAD kit (according to the manufacturer's instructions). Cell analysis was performed with a BD FACSCanto II and FlowJo software.

### Tumor-NK cell conjugate formation assay

Tumor cells and NK cells were stimulated as described above. Both cell types were stained with either Cell Tracker CMDFA or Deep Red for 30 min under serum-free conditions. Afterwards, the cells were cocultured in culture medium (target:effector cell ratio of 4:1) for 30 min at 37 °C. To prevent contact between the cells, the samples were moderately vortexed for 1 sec, and ice-cold 0.5% PFA was added. The formation of conjugates (CMFDA/Deep Red double-positive events) was immediately analyzed on a BD FACSCanto II flow cytometer via BD FlowJo software.

### Flow cytometry

Tumor cells (2×10^5^/ml) were stimulated with conditioned media for 72 h at 37 °C and 5% CO_2_. The cells were stained with fluorophore-conjugated antibodies. Isotype controls were used as negative controls. The cells were analyzed via a BD FACSCanto II flow cytometer and BD FlowJo Software. The antibodies used are listed in Table S8.

### Zymography

Tumor cells (2×10^5^/ml) were stimulated with conditioned media for 72 h at 37 °C and 5% CO2. The supernatants were collected and incubated with zymogram sample buffer at a final concentration of 80 mM Tris (pH 6.8), 1% SDS, 4% glycerol and 0.006% bromophenol blue. Proteins were separated via sodium dodecyl sulfate‒polyacrylamide gel electrophoresis (SDS‒PAGE) with 0.2% gelatin. The samples were renatured in 2.5% Triton-X-100 for 1 h at RT, and the enzymatic reaction was allowed to proceed for 16 h at 37 °C in buffer containing 50 mM Tris (pH 7.5), 200 mM NaCl, 5 mM CaCl2 and 1% Triton-X-100. To visualize the digested bands, the gel was incubated with 0.5% Coomassie blue, 30% methanol and 10% acetic acid for 2 h at RT, followed by multiple destaining steps in a mixture containing methanol and 10% acetic acid.

### Western blot

Tumor cells were stimulated with conditioned media for 72 h at 37 °C and 5% CO_2_. FaDu cells (1x10^6^ cells/sample) were lysed with buffer containing 25 mM HEPES (pH 7.3), 0.1% sodium dodecyl sulfate (SDS), 1% Triton X-100, 10 mM EDTA, 10 mM sodium pyrophosphate, 10 mM NaF, 125 mM NaCl, 1% protease inhibitor cocktail I, 1% protease inhibitor cocktail III, and 10% PhosStop. The cell debris was removed via centrifugation, and the lysates were incubated with SDS sample buffer (final concentrations of 50 mM Tris (pH 6.8), 4% glycerin, 0.8% SDS, 1.6% β-mercaptoethanol, and 0.04% bromophenol blue). The samples were boiled and analyzed via SDS‐PAGE, followed by wet transfer to PVDF membranes (Roche). The samples were incubated with primary and secondary antibodies for 2 h at RT. Chemiluminescent detection was performed with a ChemiDoc‐It imaging system (UVP, LLC, Upland, CA, USA). The antibodies used are listed in Table S9.

### Statistical analysis

Statistical analysis was performed with GraphPad Prism 8 software. The tests used in the study included one-way ANOVA, Tukey’s multiple comparisons test, and paired and unpaired *t* tests. Data are depicted as either the mean value with standard deviation (±SD) or the median, depending on the experiment.

## Supplementary information

supplementary data: figures, tables and legends

supplementary table S5

## Data Availability

The RNA-seq data generated in this study are available at GEO (GSE287001).
