# Supplementary material for: Tumor cells that resist neutrophil anticancer cytotoxicity acquire a prometastatic and innate immune escape phenotype
Source: Cell Mol Immunol. 2025 Mar 28;22(5):527–40. doi: 10.1038/s41423-025-01283-w (PMC12041228; doi:10.1038/s41423-025-01283-w)
Supplement: Supplementary file 1 — supplementary data: figures, tables and legends [file 41423_2025_1283_MOESM1_ESM.docx]

**Supplemental Figures**


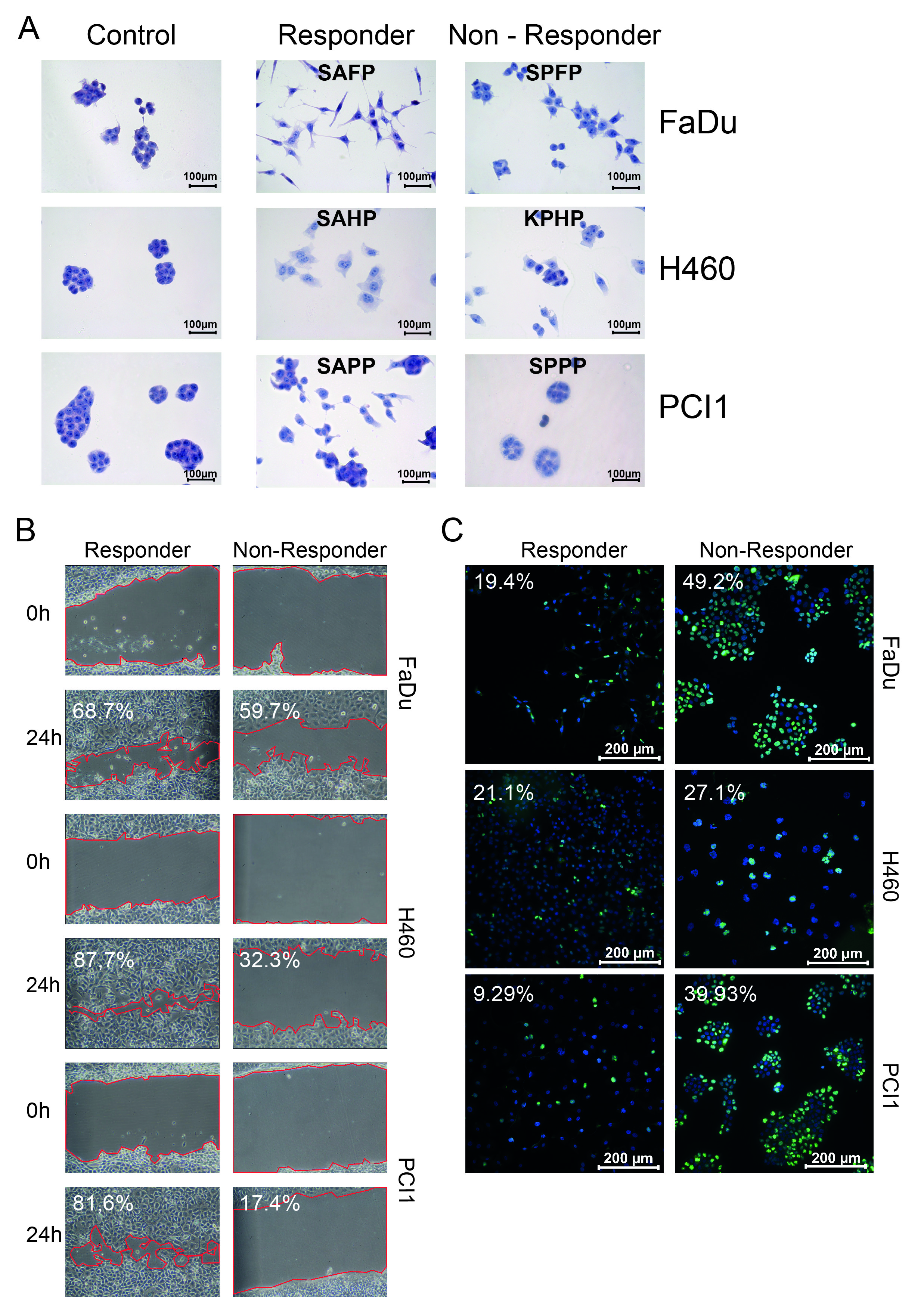


**Fig. S1 Induction of a mesenchymal morphotype in HNC and lung cancer cell lines by different bacterial strains**

A description of the supernatants can be found in Table S3. Figure shows one representative experiment (out of three performed with similar results). **(A)** Hematoxylin staining of tumor cells stimulated with supernatants for 72 h. **(B)** Tumor cells treated with supernatants were grown until confluence. Closure of a scratch “wound” was recorded after 24 h. Percentage of the scratch closure was quantified with ImageJ. **(C)** Expression of Ki67 was analyzed by immunofluorescence in stimulated tumor cells. Percentage of Ki67 positive cells was obtained with ImageJ.


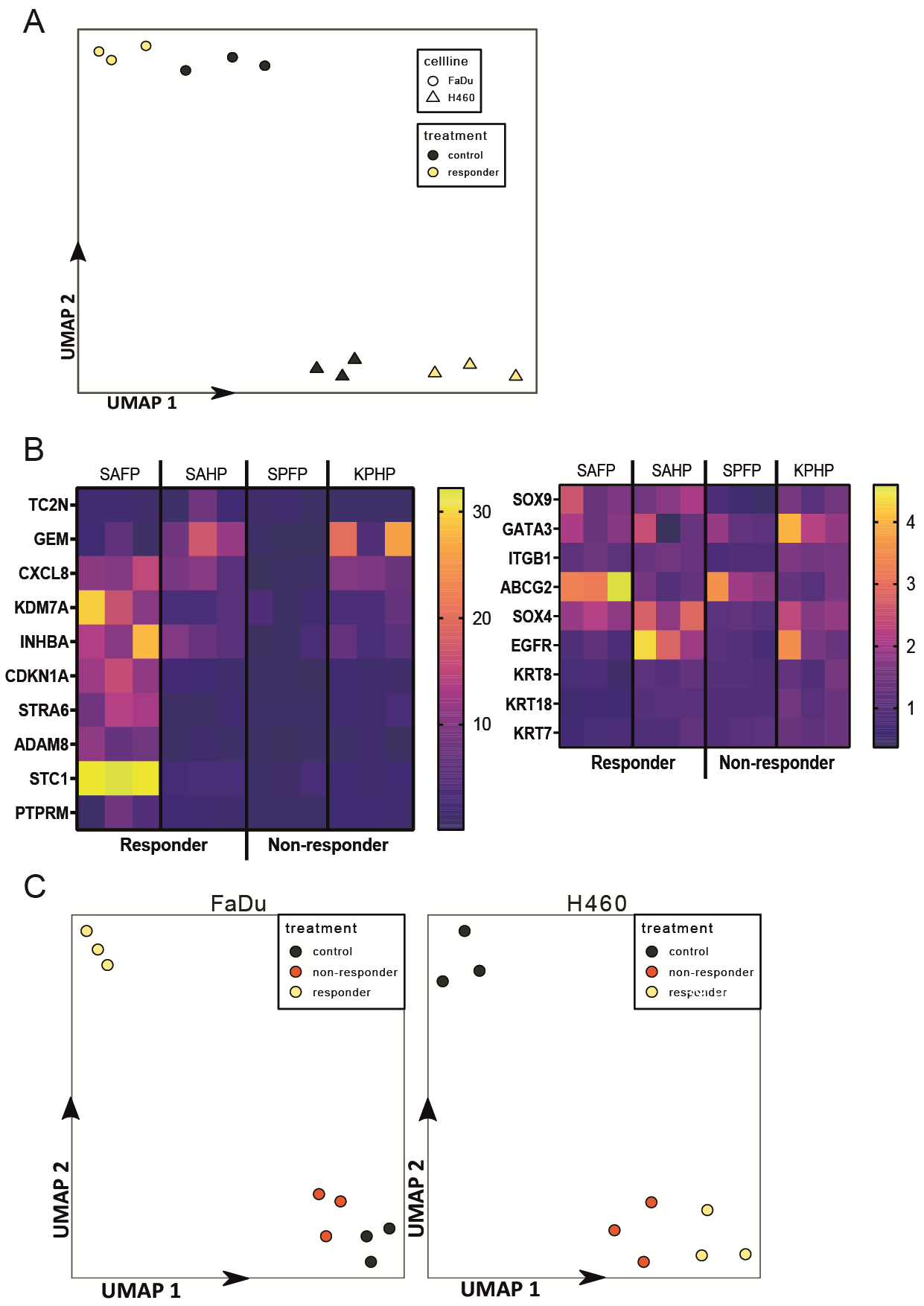


**Fig. S2 Differences in gene expression patterns between cell lines and stimulation conditions**

**(A)** UMAP analysis of all expressed genes in FaDu and H460 cells. The shape of points indicates the cell line (circle = FaDu, triangle = H460), the color discriminates the response type/treatment (black = control, yellow = responder). **(B)** Heatmaps showing metastasis-linked genes in both cell lines, comparing responders (left) and non responders (right). Gene expression was normalized to the control. **(C)** UMAP analysis of metastasis-linked genes separated for the two cell lines FaDu (left) and H460 (right), colored by response type/treatment (black = control, red = non-responder, yellow = responder).


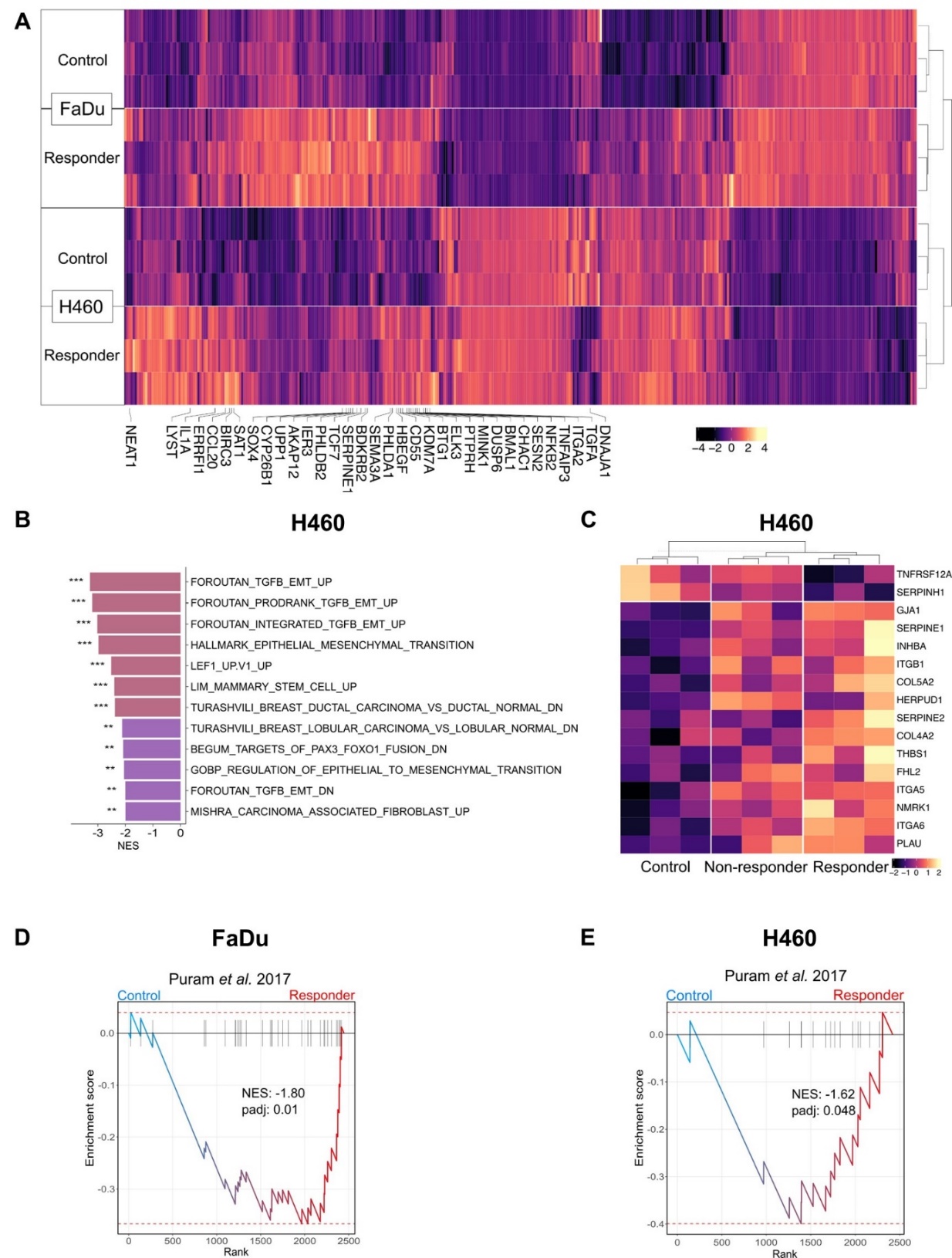


**Fig. S3 Bulk RNAseq of control, non-responder and responder samples from FaDu and H460 cells.** **(A)** Heatmap of differentially expressed genes between control and responder samples from the list of selected genes shown in Table S4, linked to metastasis. Indicated genes had a p-adjusted value < 0.001. A selection of these genes with a p-adjusted value < 0.05 is shown in Figure 2B. **(B)** Gene set enrichment analysis (GSEA) is shown for differentially expressed genes between control and responder samples from H460 for all gene sets containing the wording 'EMT'. All gene sets with a p-adjusted value < 0.01 were enriched in responder samples. There were no pathways enriched in the control samples. **(C)** Heatmap of differential expressed genes between control and responder H460 samples with a p-adjusted value < 0.1. Genes comprise p-EMT genes from (Ref. 47). **(D-E)** Corresponding GSEA plots for p-EMT genes described by (Ref. 47) using differentially expressed genes between control and responder FaDu **(D)** and H460 **(E)** samples. NES and padj (p-adjusted) values for the specific pathway are indicated on each plot.

**
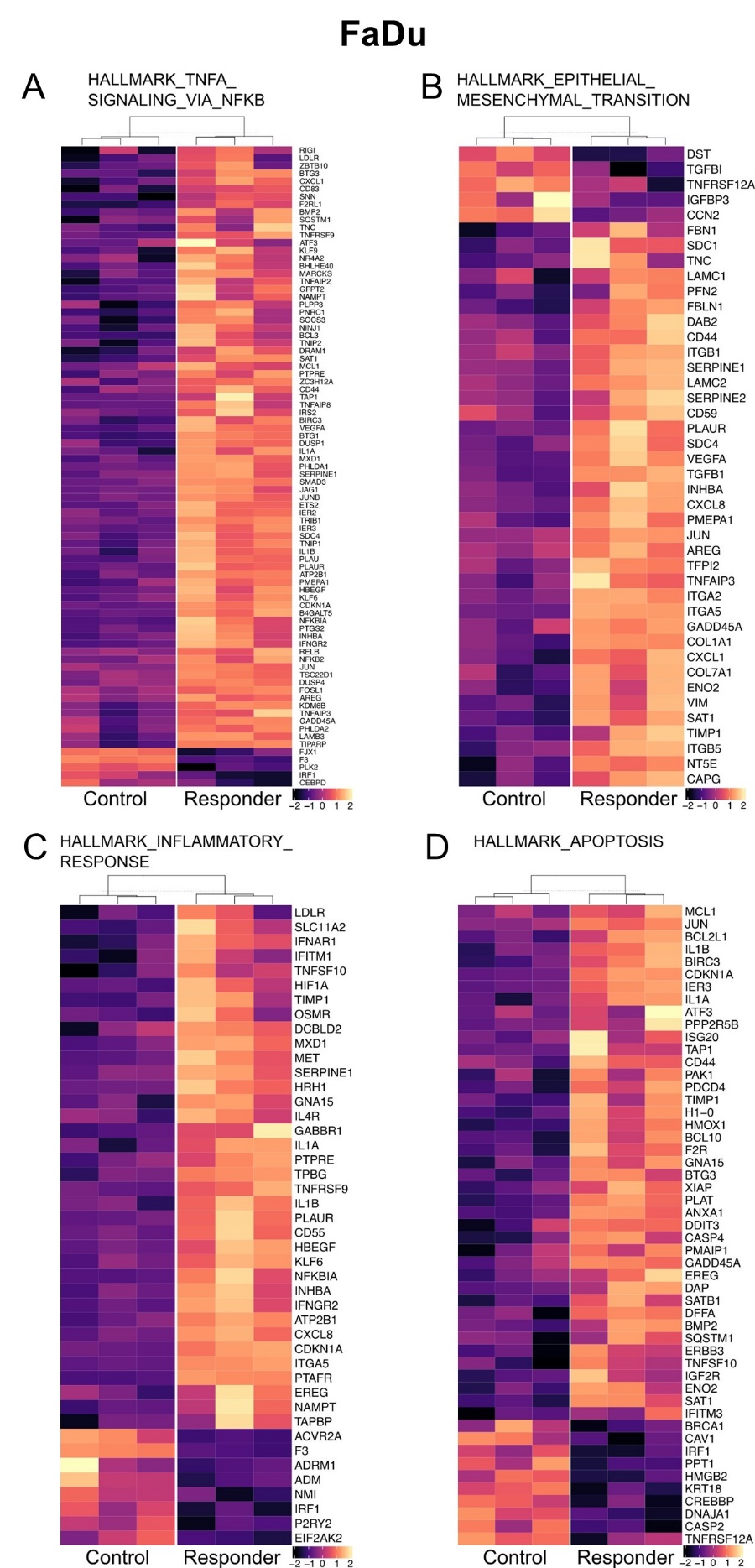
**

**Fig. S4 Heatmaps of differential expressed genes between control and responder FaDu samples for selected pathways.** **(A-D)** Related to Fig. 2E, all genes from the corresponding pathways with a p-adjusted value < 0.1 are shown: Hallmark TNF signaling via NFκB (A), Hallmark EMT (B), Hallmark inflammatory response (C) and Hallmark Apoptosis (D).

**
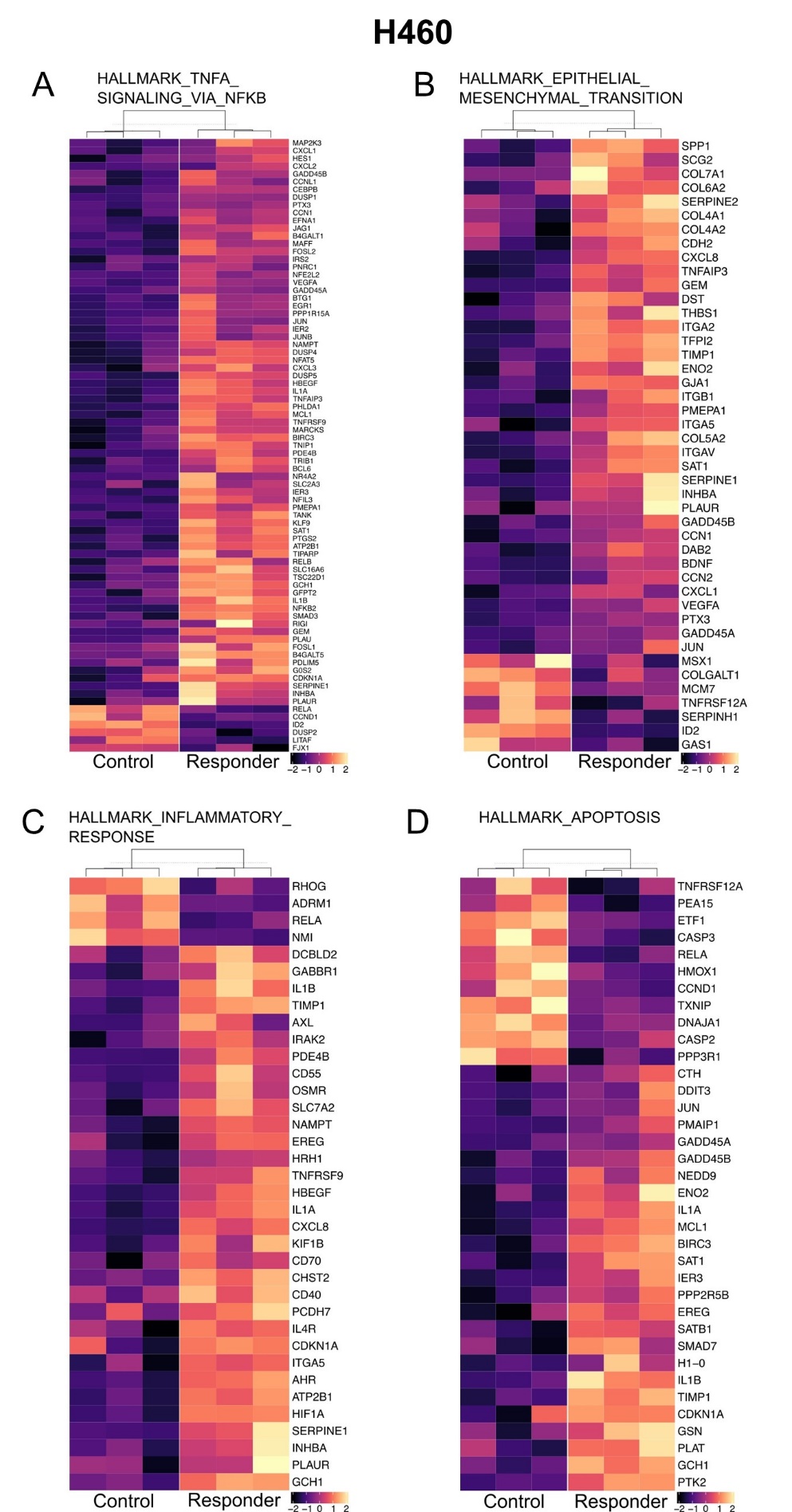
**

**Fig. S5 Heatmaps of differential expressed genes between control and responder H460 samples for selected pathways.** **(A-D)** Related to Fig. 2F, all genes from the corresponding pathways with a p-adjusted value < 0.1 are shown: Hallmark TNF signaling via NFκB (A), Hallmark EMT (B), Hallmark inflammatory response (C) and Hallmark Apoptosis (D).


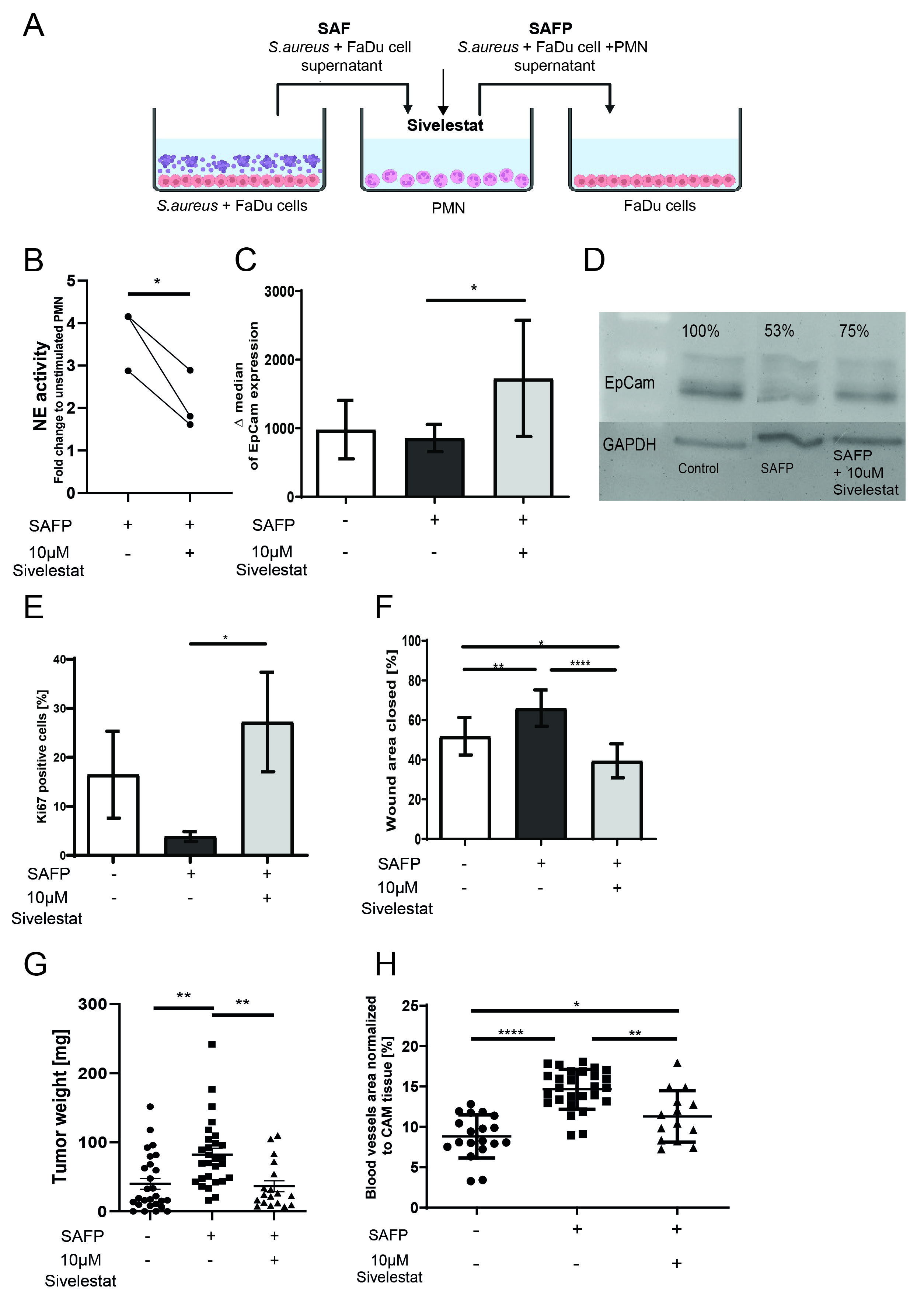


**Fig. S6 Role of neutrophil elastase in tumor cell stimulation. (A)** Sivelestat (NE inhibitor, 10 µM) was added during production of SAFP supernatant as indicated in panel A. **(B)** Activity of NE was determined in the supernatant, in the presence or absence of Sivelestat, using the NE activity assay, n=3. **(C-F)** Tumor cells were stimulated for 72 h with SAFP that was generated with or without Sivelestat. The expression of EpCAM was determined by flow cytometry, n=3 **(C)**, and Western Blot **(D)**. One representative image is shown out of 3 experiments. **(E)** Frequency of Ki67-positive tumor cells were quantified by immunofluorescence staining; n=3. **(F)** Scratch closure was determined by scratch assay, n=7. **(G-H)** Effects of Sivelestat were analyzed in the *in ovo* CAM assay, determining tumor weight **(G)** and angiogenesis in the upper CAM **(H)** (control n=20, SAFP n=27, SAFP+Sivelestat n=15). **(B-F)** n indicates number of independent experiments, **(G-H)** n indicates number of eggs used in experiment. Statistical analysis was performed with an unpaired t-test **(B, C, E, F)** and one-way ANOVA test with Tukey's multiple comparisons test **(G, H)**: *=p<0.05, ** p<0.01, **** p≤0.0001. Data are displayed as mean +/- SD.


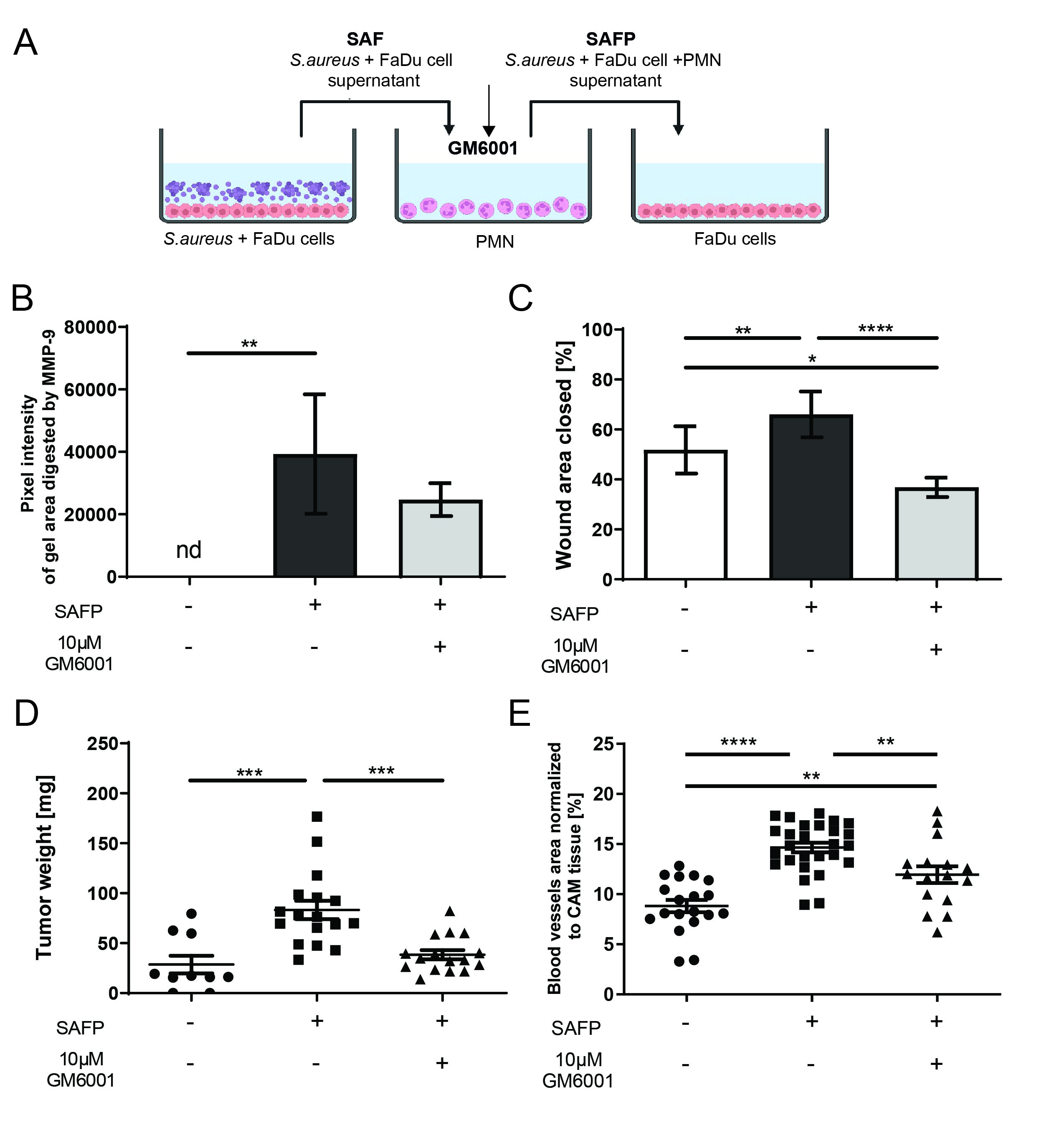


**Fig. S7 Role of MMP-9 in tumor cell stimulation. (A)** GM6001 (MMP-9 inhibitor, 10 µM) was added during the production of SAFP supernatant as indicated in panel A. **(B)** Levels of active MMP-9 in PMN supernatants were determined by zymography, n=3. **(C-E)** Tumor cells were stimulated for 72 h with SAFP that was generated with or without GM6001. Effects of GM6001 on scratch closure (**C**; n=4); tumor growth in the *in ovo* CAM assay (**D**, control n=10, SAFP n=17, SAFP+GM6001 n=16) and angiogenesis in the upper CAM (**E**, control n=19, SAFP n=27, SAFP+GM6001 n=16) were determined. **(B)** n indicates number of independently acquired supernatants, **(C)** n indicates number of independent experiments, **(D-E)** n indicates number of eggs used in the experiment. Statistical analysis was performed with an unpaired t-test **(B, C)** and one-way ANOVA test with Tukey's multiple comparisons test **(D, E)**: *=p<0.05, ** p<0.01, *** p<0,001, **** p≤0.0001. Data are displayed as mean +/- SD.

**Supplemental Tables**

**Table S1. Characteristics of patients used for tumor tissue study cohort 1.**

| **Patients’ characteristics** | **Numbers** | **% of total** |
| --- | --- | --- |
| **Gender** |  |  |
| Male | 41 | 75.9 |
| Female | 13 | 24.1 |
|  |  |  |
| **Tumor localization** |  |  |
| Oropharynx | 52 | 96.3 |
| Oral cavity | 2 | 3.7 |
|  |  |  |
| **Tumor size** |  |  |
| T1 | 1 | 1.9 |
| T2 | 16 | 29.6 |
| T3 | 25 | 46.3 |
| T4a/b | 12 | 22.2 |
|  |  |  |
| **Lymph node metastasis** |  |  |
| N0 | 17 | 31.5 |
| N1 | 10 | 18.5 |
| N2a | 4 | 7.4 |
| N2b | 10 | 18.5 |
| N2c | 12 | 22.2 |
| N3 | 1 | 1.9 |
|  |  |  |
| **Distant metastasis** |  |  |
| M0 | 50 | 92.6 |
| M1 | 4 | 7.4 |
|  |  |  |
| **HPV16 status** |  |  |
| Positive | 21 | 38.9 |
| Negative | 33 | 61.1 |
|  |  |  |
| **Histological grade** |  |  |
| Grade 1 | 5 | 9.3 |
| Grade 2 | 31 | 57.4 |
| Grade 3 | 18 | 33.3 |

**Table S2. Characteristics of patients used for tumor tissue study cohort 2.**

| **Patients’ characteristics** | **Numbers** | **% of total** |
| --- | --- | --- |
| **Gender** |  |  |
| Male | 40 | 61.5 |
| Female | 25 | 38.5 |
|  |  |  |
| **Tumor localization** |  |  |
| Oropharynx | 51 | 78.5 |
| Oral cavity | 14 | 21.5 |
|  |  |  |
| **Tumor size** |  |  |
| T1 | 8 | 12.3 |
| T2 | 22 | 33.8 |
| T3 | 20 | 30.8 |
| T4a/b | 15 | 23.1 |
|  |  |  |
| **Lymph node metastasis** |  |  |
| N0 | 21 | 32.3 |
| N1 | 17 | 26.2 |
| N2a | 5 | 7.7 |
| N2b | 5 | 7.7 |
| N2c | 12 | 18.4 |
| N3 | 5 | 7.7 |
|  |  |  |
| **Distant metastasis** |  |  |
| M0 | 64 | 98.5 |
| M1 | 1 | 1.5 |
|  |  |  |
| **Histological grade** |  |  |
| Grade 1 | 1 | 1.5 |
| Grade 2 | 26 | 40 |
| Grade 3 | 18 | 27.7 |
| unknown | 20 | 30.8 |

**Table S3 Detailed description of supernatants**

| **Abbreviation** | **Bacteria strain** | **Tumor cell line** | **PMN** |
| --- | --- | --- | --- |
| F* | *-* | FaDu | - |
| FP* | *-* | FaDu | PMN |
| SAF* | *S. aureus* | FaDu | - |
| SAFP* | *S. aureus* | FaDu | PMN |
| SPF | *S. pneumoniae* | FaDu | - |
| SPFP | *S. pneumoniae* | FaDu | PMN |
| H | *-* | H460 | - |
| HP | *-* | H460 | PMN |
| SAH | *S. aureus* | H460 | - |
| SAHP | *S. aureus* | H460 | PMN |
| KPH | *K. pneumoniae* | H460 | - |
| KPHP | *K. pneumoniae* | H460 | PMN |
| P | *-* | PCI1 | - |
| PP | *-* | PCI1 | PMN |
| SAP | *S. aureus* | PCI1 | - |
| SAPP | *S. aureus* | PCI1 | PMN |
| SPP | *S. pneumoniae* | PCI1 | - |
| SPPP | *S. pneumoniae* | PCI1 | PMN |

Supernatants were produced in two steps: First, tumor cells (column 3) were stimulated using one bacteria strain (column 2) and the following supernatants were collected (Abbreviation: Bacterial strain and tumor cell line; SAF, SPF, SAH, KPH, SAP, SPP). In the next step, part of this supernatant was directly used for the stimulation of PMN (P) and the following supernatants were generated (Abbreviation: Bacterial strain, tumor cell line and PMN; SAFP, SPFP, SAHP, KPHP, SAPP, SPPP. FP for instance constitutes the supernatant of PMN (P) stimulated by FaDu tumor cell supernatant (F). ***F, FP, SAF and SAFP generation is exemplarily shown in Fig 1A.**

**Table S4 List of genes selected for metastasis-related RNAseq analysis**

| ABCG2 | CCL20 | CYP1A1 | FN1 | INHBA | MAST4 | PIK3CA | SDC4 | TGFA |
| --- | --- | --- | --- | --- | --- | --- | --- | --- |
| ABLIM1 | CCM2 | CYP1B1 | FN1 | ITGA2 | MDGA1 | PIK3CD | SELENBP1 | TGFB1 |
| AC007686.4 | CCND1 | CYP26B1 | FNDC3B | ITGA5 | MET | PIK3R1 | SEMA3A | THBD |
| ADAM8 | CCND2 | DAB2 | FOS | ITGA6 | METTL27 | PIM1 | SEMA3B | THBS1 |
| ADORA2B | CCNE2 | DAPP1 | FOSL1 | ITGB1 | MINK1 | PKP2 | SERPINA1 | THY1 |
| AEN | CD24 | DDIT4 | FUT1 | JAG1 | MMP1 | PLAK | SERPINB1 | TIMP1 |
| AGRN | CD44 | DDR1 | FUT3 | JDP2 | MMP13 | PLAU | SERPINE1 | TJP1 |
| AHR | CD49C | DDR2 | FYN | JUN | MMP3 | PLAUR | SERPINE2 | TLR6 |
| AJUBA | CD55 | DGKE | GAB2 | KCNG1 | MMP9 | PLEK2 | SESN2 | TMEM156 |
| AKAP12 | CD82 | DNAJA1 | GADD45A | KCNN3 | MMRN1 | PLK2 | SH2B2 | TNFAIP3 |
| AKNA | CDC25A | DSP | GALNT6 | KDM4A | MPZL2 | PLOD1 | SH2B3 | TNF-A |
| AL137003.2 | CDCP1 | DUSP1 | GAP43 | KDM6B | MRAS | PLOD2 | SIAH2 | TNFRSF21 |
| ALDH1A1 | CDH1 | DUSP2 | GATA3 | KDM7A | MTSS1 | PMAIP1 | SLC1A4 | TNFRSF8 |
| ALDH1A3 | CDH11 | DUSP4 | GCH1 | KDM7A-DT | MXD1 | PPIF | SLC29A3 | TNFRSF9 |
| ALDH6A1 | CDH2 | DUSP5 | GEM | KIF5B | MYADM | PPP1R15A | SLC38A5 | TNFSF9 |
| AMOTL2 | CDK12 | DUSP6 | GINS3 | KLF6 | MYC | PRDM1 | SLC7A11 | TP53 |
| ANTXR2 | CDKN1A | DUSP7 | GJB3 | KLHDC8B | MYORG | PRKCE | SLC8B1 | TRIB1 |
| ANXA4 | CDKN2A | ECM1 | GJB5 | KRT15 | NAV2 | PSMB9 | SMPD1 | TRIB2 |
| APOC1 | CEBPB | EGFR | GNA15 | KRT16A | NAV3 | PTAFR | SNAI1 | TRIB3 |
| AQP3 | CEBPG | EGR1 | GSDMB | KRT17 | NDRG1 | PTEN | SNAI2 | TRIM35 |
| AREG | CES1 | ELK3 | GST-PHI | KRT18 | NEAT1 | PTGS1 | SNPH | TRMU |
| ARNTL | CFD | ELMO1 | H19 | KRT7 | NES1 | PTGS2 | SOCS3 | TSC22D3 |
| ARSD | CGA | EPCAM | HBEGF | KRT8 | NF1 | PTPRG | SOX4 | TUBA1A |
| ASNS | CHAC1 | EPHA2 | HIF1A | KYNU | NFKB2 | PTPRH | SOX9 | TUFT1 |
| ATF3 | CHRM3 | ERBB2 | HMOX1 | LAD | NFKBIA | PTPRM | SP140 | TWIST1 |
| ATF5 | CLDN1 | ERBB4 | HRAS | LAMA3 | NKX3-1 | PTX3 | SPP1 | UBASH3B |
| ATP8A1 | CLDN11 | EREG | HS3ST1 | LAMB2 | NOTCH1 | RAC2 | SPRY4 | UCHL1 |
| ATXN1 | CLDN4 | ERN1 | HSPA1A | LAMC2 | NOTCH2 | RAET1E | SRGN | UGCG |
| BBC3 | CLDN7 | ERRFI1 | HSPA1B | LATS2 | NOTCH2NLA | RARB | STC1 | ULBP2 |
| BDKRB2 | CLIP2 | ETS1 | HYAL1 | LIMA1 | NR1D1 | RASAL2 | STC2 | ULK4 |
| BDNF | COL10A1 | ETV5 | ID2 | LIN7A | NR1D2 | RASGEF1A | STEAP1 | UNG |
| BEND7 | COL1A2 | EXO1 | IER3 | LMO4 | NRCAM | RASSF2 | STK40 | UPP1 |
| beta-NGF | COL4A5 | EYA4 | IGF1R | LPAR3 | NTSR1 | RELB | STRA6 | VASH1 |
| BIRC3 | COL6A3 | F2R | IGFBP-5 | LRBA | NUAK2 | RHOB | STX1A | VAV3 |
| BIRC7 | COL7A1 | FBLN2 | IGFBP-6 | LRFN4 | NXN | RIPK2 | SUCNR1 | VEGFA |
| BMI1 | CORO6 | FBN1 | IGFN1 | LRRC19 | PAX6 | RND3 | SYNGR3 | VEGF-C |
| BMP2 | COX19 | FCGR2A | IGSF3 | LURAP1L | PCDH17 | RPS6KA2 | TC2N | VIM |
| BTG1 | CRCP | FGF | IL11 | LYPD3 | PDE4D | RRM2 | TCF7 | WNT5A |
| C5AR1 | CSRNP1 | FGF5 | IL1A | LYST | PDGFRA | RSPH3 | TENASCIN-C | WSCD1 |
| CAK | CSRP2 | FGFR1 | IL1B | MAFF | PDLIM4 | S100A14 | TFEB | XBP1 |
| CARD10 | CTNNB1 | FGFR2 | IL1R1 | MAN1A1 | PGF | SAT1 | TFPI2 | ZEB1 |
| CBLB | CXCL5 | FGFR3 | IL1RL1 | MAP1B | PHLDA1 | SCIN | TFRC | ZEB2 |
| CCDC33 | CXCL8 | FLNA | IL24 | MAP3K5 | PHLDB2 | SDC1 | TGASE | ZNF114 |

List of 384 genes that were reported to be linked to metastasis, manually curated from literature research by the authors. Exact values for log2foldchange and padj are shown in a separate excel spreadsheet designated Table S5.

Table S5. Uploaded as separate file in excel format.

Table S6: Sequence and annealing temperatures for oligonucleotides used in this study

| **Gene** | **Forward primer** | **Reverse primer** | **T_m_** |
| --- | --- | --- | --- |
| β-actin | AGCGGGAAATCGTGCGTG | GGGTACATGGTGGTGCCG | 60°C |
| MMP-9 | CTATGTACCGCTTCACTGAGGG | GACTCAAAGGCACAGTAGTGGC | 60°C |
| N-Cadherin | CCTCCAGAGTTTACTGCCATGAC | GTAGGATCTCCGCCACTGATTC | 60°C |
| SNAI1 | AATCGGAAGCCTAACTACAGCGAG | CCTTGGCCTCAGAGAGCTGG | 58°C |
| TCF-8 | GGCATACACCTACTCAACTACGG | TGGGCGGTGTAGAATCAGAGTC | 60°C |

Table S7: Antibodies for immunofluorescence staining of PMN, tumor cells and HNC tissue samples

| Antibody | Isotype | Clone | Source | Identifier | Dilution |
| --- | --- | --- | --- | --- | --- |
| Mouse anti-human NE | IgG2a | 240 | Abcam | Cat#: ab20800  RRID: AB_44583 | 1:100 |
| Rabbit anti-human MMP9 | IgG | D6O3H | Cell Signaling Technology | Cat#: 13667 RRID:AB_2798289 | 1:100 |
| Alexa Fluor® 647 mouse anti-human CD66b | IgG1 | 6/40c | BioLegend | Cat#:392912 RRID:AB_2814339 | 1:100 |
| Alexa Fluor® 594 mouse anti-human CD66b | IgG1 | 6/40c | BioLegend | Cat#:392908 RRID:AB_2801026 | 1:100 |
| FITC mouse anti-human CD66b | IgG1 | 80H3 | Beckmann coulter | Cat# IM0531U RRID: AB_10638220 | 1:100 |
| Mouse anti-human Ki67 | IgG1 | MIB-1 | Agilent Dako | Cat#:M7240  RRID:AB_2631211 | 1:100 |
| Chicken anti-Vimentin | IgY | Polyclonal | Novus | Cat#: NB300-223  RRID:AB_10003206 | 1:100 |
| Alexa Fluor® 488 mouse anti-human PanCytokeratin | IgG1 | AE1/AE3 | Thermo Fisher Scientific, eBioscience | Cat#: 53-9003-82  RRID: AB_1834350 | 1:500 |
| Mouse anti-human Vimentin | IgM | VIM 13.2 | Sigma-Aldrich | Cat#: V5255  RRID: AB_477625 | 1:400 |
| Mouse anti-human MPO | IgG1 | 2C7 | Bio-Rad | Cat#: MCA1757  RRID: AB_2146467 | 1:1000 |
| Rabbit anti-human Histone H3 (citrulline R2+R8+R17) | IgG | Polyclonal | Abcam | Cat#: Ab5103  RRID: AB_304752 | 1:1000 |
| Alexa Fluor™ 480 Donkey anti-mouse | IgG | Polyclonal | Thermo Fisher Scientific, Molecular Probes | Cat# A-21202  RRID:AB_141607 | 1:200 |
| Alexa Fluor™ 790 Donkey anti-mouse | IgG | Polyclonal | Jackson ImmunoResearch Labs, Dianova | Cat#: 705-655-147  RRID:AB_2340441 | 1:200 |
| Alexa Fluor™ 546 Donkey anti-rabbit | IgG | Polyclonal | Thermo Fisher Scientific, Invitrogen | Cat#: A10040  RRID: AB_2534016 | 1:200 |
| Alexa Fluor™ 647 Donkey anti-chicken | IgY | Polyclonal | Jackson ImmunoResearch Labs, Dianova | Cat#: 703-606-155  RRID:AB_2340380 | 1:200 |
| Cy3 Donkey anti-mouse | IgM | Polyclonal | Jackson ImmunoResearch Labs, Dianova | Cat#: 715-166-020  RRID:AB_2340815 | 1:600 |

Table S8: Antibodies for flow cytometry

| Antibody | Isotype | Clone | Source | Identifier | Dilution |
| --- | --- | --- | --- | --- | --- |
| PE Mouse anti-human EpCAM/Trop1(CD326) | IgG1 | IB7 | Thermo Fisher Scientific, eBioscience | Cat#: 14-9326-82,  RRID: AB_795876 | 1:500 |
| PE Mouse anti-human IgG1 | Control | P3.6.2.8.1 | Thermo Fisher Scientific, eBioscience | Cat#:12-4714-82  RRID: AB_470060 | 1:200 |

Table S9: Antibodies for western blot

| Antibody | Isotype | Clone | Source | Identifier | Dilution |
| --- | --- | --- | --- | --- | --- |
| Rabbit anti-human EpCAM | IgG | D4K8R | Cell Signaling Technology | Cat#: 36746  RRID: AB_2799105 | 1:1000 |
| Rabbit anti-human GAPDH | IgG | 14C10 | Cell Signaling Technology | Cat#: 2118  RRID: AB_1031003 | 1:5000 |
| Goat anti-rabbit HRP-linked | IgG | Polyclonal | Cell Signaling Technology | Cat#: 7074  RRID: AB_2099233 | 1:3000 |
